# Identification and expression analysis of *EDR1*-like genes in tobacco (*Nicotiana tabacum*) in response to *Golovinomyces orontii*

**DOI:** 10.7717/peerj.5244

**Published:** 2018-07-10

**Authors:** Lei Wu, Xiaoying Zhang, Bingxin Xu, Yueyue Li, Ling Jia, Rengang Wang, Xueliang Ren, Genhong Wang, Qingyou Xia

**Affiliations:** 1 State Key Laboratory of Silkworm Genome Biology, Southwest University, Chongqing, China; 2 Molecular Genetics Key Laboratory of China Tobacco, Guizhou Academy of Tobacco Science, Guiyang, China

**Keywords:** Powdery mildew, *ENHANCED DISEASE RESISTANCE1*, Tobacco, Phylogenetic analysis, Expression patterns

## Abstract

*ENHANCED DISEASE RESISTANCE1* (*EDR1*) encodes a *Raf*-like mitogen-activated protein kinase, and it acts as a negative regulator of disease resistance and ethylene-induced senescence. Mutations in the *EDR1* gene can enhance resistance to powdery mildew both in monocotyledonous and dicotyledonous plants. However, little is known about *EDR1*-like gene members from a genome-wide perspective in plants. In this study, the tobacco *(Nicotiana tabacum)* *EDR1*-like gene family was first systematically analyzed. We identified 19 *EDR1*-like genes in tobacco, and compared them to those from Arabidopsis, tomato and rice. Phylogenetic analyses divided the *EDR1*-like gene family into six clades, among them monocot and dicot plants were respectively divided into two sub-clades. *NtEDR1-1A* and *NtEDR1-1B* were classified into clade I in which the other members have been reported to negatively regulate plant resistance to powdery mildew. The expression patterns of tobacco *EDR1*-like genes were analyzed after plants were challenged by *Golovinomyces orontii*, and showed that several other *EDR1*-like genes were induced after infection, as well as *NtEDR1-1A* and *NtEDR1-1B*. Expression analysis showed that *NtEDR1-13* and *NtEDR1-16* had exclusively abundant expression patterns in roots and leaves, respectively, and the remaining *NtEDR1*-like members were actively expressed in most of the tissue/organ samples investigated. Our findings will contribute to further study of the physiological functions of *EDR1*-like genes in tobacco.

## Introduction

Powdery mildew, an important fungal disease in agriculture and horticulture, is caused by ascomycetes of the order Erysiphales (Dean et al., 2012; Glawe, 2008). Worldwide, powdery mildews colonize a wide variety of plant species—over 650 monocots and over 9,000 dicots (Schulzelefert & Vogel, 2000). They cause significant harvest losses in crops such as wheat, barley and tomato (Dean et al., 2012), ornamental plants such as roses (Horst, Kawamoto & Porter, 1992; Linde et al., 2006; Palmer & Henneberry, 1960) and fruits like grapevine (Donald et al., 2002). The current universal method for controlling powdery mildew is by application of fungicide, but this can have a serious environmental impact. Research has detected high concentrations of residual fungicides in food and crops. These chemicals can reach human cells via the food chain and negatively affect cellular metabolism (Pirozzi et al., 2016; Yang et al., 2018). Thus, identifying genes with functions in fundamental plant defense may have potential for reducing production losses caused by powdery mildew. In 1998, Frye and Innes found an Arabidopsis mutant that displayed *ENHANCED DISEASE RESISTANCE1* (*EDR1*) to *Pseudomonas syringae* and *Golovinomyces cichoracearum* (formerly named *Erysiphe cichoracearum*) (Frye & Innes, 1998). Functional studies of *EDR1* genes have been reported in Arabidopsis, wheat, rice and tomato (Frye & Innes, 1998; Gao et al., 2015; Shen et al., 2011; Zhang et al., 2017).

In Arabidopsis, *AtEDR1* encodes *Raf*-like mitogen-activated protein kinase. Loss-of-function mutants of *EDR1* are resistant to the biotrophic pathogens *G. cichoracearum* (Frye & Innes, 1998) and *Hyaloperonospora arabidopsidis* (van Hulten et al., 2006) and also show enhanced susceptibility to hemibiotrophic *Colletotrichum higginsianum* and necrotrophic *Alternaria brassicicola* (Hiruma et al., 2011). Thus, *EDR1* mutants can obtain broad-spectrum resistance. In wheat, knockdown *TaEDR1* mutants from VIGS or RNAi showed increased resistance to virulent isolates of *Blumeria graminis f.* sp. *tritici* (Zhang et al., 2017). Additionally, wheat *EDR1* plants generated by simultaneous modification of the three homologs of *TaEDR1* with CRISPR/Cas9 (clustered regularly interspaced short palindromic repeats/CRISPR-associated) technology, did not exhibit powdery mildew-induced cell death (Zhang et al., 2017). The sequence ortholog of Arabidopsis *EDR1* in rice, *OsEDR1*, has a negative function in the defense response (Shen et al., 2011). The expression of *OsEDR1* was induced by wounding, jasmonic acid, salicylic acid, ethylene, abscisic acid, hydrogen peroxide, fungal elicitor chitosan, drought, high salt, sugar and heavy metals (Kim et al., 2003). The RNAi plants or T-DNA insertion mutants of *OsEDR1* had enhanced resistance to bacterial pathogen *Xanthomonas oryzae* pv. *oryzae* (Shen et al., 2011). However, silencing of two *EDR1* homologs (Solyc01 g097980 and Solyc06 g068980) separately did not confer significant resistance against *Oidium neolycopersici* in tomato (Gao et al., 2015). Whether simultaneous knockdown of these two homologs could enhance powdery mildew resistance needs further investigation.

The *EDR1* protein consists of an N-terminal domain of unknown function and a C-terminal kinase domain (Tang & Innes, 2002). The C-terminal of *EDR1* encodes a serine/threonine-protein kinase with homology to CONSTITUTIVE TRIPLE RESPONSE1 (Tang, Christiansen & Innes, 2005), a negative regulator of ethylene responses (Cao et al., 1997; Kieber et al., 1993). The N-terminal regulatory domain of *EDR1* could interact with MKK4 and MKK5 to negatively regulate the MKK4/MKK5–MPK3/MPK6 kinase cascade pathway (Zhao et al., 2014). It was also reported that *EDR1* could act as a suppressor of disease resistance and programmed cell death (for both abiotic and biotic stresses) by adjusting signal processing through the hormone-mediated pathways of salicylic acid, ethylene and abscisic acid (Frye & Innes, 1998; Tang, Christiansen & Innes, 2005), and as a positive regulator of expression of plant defensins (Hiruma et al., 2011).

Although the *EDR1* pathway is highly conserved in crop plants (Frye, Tang & Innes, 2001), whether one *EDR1* plays a main role or several *EDR1* homologs work together to function as negative regulators of plant defense needs further detailed analysis for a specific species. For example, in tomato, RNAi of the two *EDR1* homologs separately did not produce significant resistance to *O. neolycopersici* (Gao et al., 2015). It also revealed that knockout of three *EDR1* homologs in wheat did not produce complete resistance (Zhang et al., 2017). Thus, complete and systematic study of the *EDR1*-like genes is needed and will help to find potential target genes for plant breeding. This study involved the first systematic identification and analysis of *EDR1*-like genes in plants. We aim to provide useful information for further exploring the physiological function of *EDR1*-like genes in tobacco and other plant species.

## Materials and Methods

### Identification of the *EDR1*-like gene family

The genome, gene and protein sequences of Arabidopsis, tomato and rice were downloaded from PlantGDB (http://www.plantgdb.org/), and those of tobacco from the Sol Genomics Network (http://solgenomics.net/organism/). To identify *EDR1*-like genes in the above-mentioned species, two *EDR1* proteins from Arabidopsis (GenBank accession: ABR45974.1) and wheat (GenBank accession: AAU89661.2) were used as the query subjects in a reciprocal Basic Local Alignment Search Tool Protein (BLASTP) analysis with *e*-values < 1E-50. Then, the online software SMART (http://smart.embl-heidelberg.de/) was used to identify the predicted *EDR1*-like protein domains. Conserved *EDR1* domains and kinase domains within the acquired *EDR1* sequences were confirmed by searching NCBI’s conserved domain database (http://www.ncbi.nlm.nih.gov/Structure/cdd/docs/cdd_search.html).

### Phylogenetic analysis and conserved domain detection

Phylogenetic trees were constructed in MEGA Version 7.0 (Kumar, Stecher & Tamura, 2016), using the Neighbor-Joining (NJ) method (Saitou, 1987) with parameters of pairwise gap deletion and 1,000 bootstraps. Multiple sequence alignment of the amino acid sequences of *EDR1*-like proteins was performed using the ClustalX program (version 1.83) (Thompson et al., 1997) and GeneDoc. Maximization for Motif Elicitation program (MEME, http://alternate.meme-suite.org) (Bailey et al., 2006) was used to predict the conserved motif of *EDR1*-like proteins in *N. tabacum*.

### Gene structure and bioinformatic analysis of tobacco *EDR1*-like genes

The genomic structure of the *EDR1*-like gene family was analyzed using GSDS 2.0 (http://gsds.cbi.pku.edu.cn/) (Hu et al., 2015). Protparam (http://web.expasy.org/protparam/) was used to analyze the basic physical and chemical properties of tobacco *EDR1*-like genes. Subcellular localization of the *EDR1*-like genes was determined using online software Plant-mPLoc (http://www.csbio.sjtu.edu.cn/bioinf/plant-multi/) (Chou & Shen, 2008, 2010a, 2010b). The nuclear localization signals (NLS) sequences were predicted by cNLS Mapper (http://nls-mapper.iab.keio.ac.jp/cgi-bin/NLS_Mapper_form.cgi#opennewwindow) (Kosugi et al., 2009a, 2009b). Transmembrane helices were predicted using OCTOPUS (http://octopus.cbr.su.se/index.php#opennewwindow) (Viklund & Elofsson, 2008). The three-dimensional (3D) structures were predicted using the online I-TASSER program (http://zhanglab.ccmb.med.umich.edu/I-TASSER/) (Zhang, 2008). The confidence score (C-score) was used to estimate the model’s global accuracy in I-TASSER (Yang & Zhang, 2015), and high C-score indicates a high-quality structure prediction (Roy, Kucukural & Zhang, 2010). The model with highest C-score in all five prediction models was further used to construct 3D model of the target genes. The constructed 3D model was examined and visualized using Chimera 1.2 (https://www.cgl.ucsf.edu/chimera/).

### Plant materials and stress treatments

Common tobacco (*Nicotiana tabacum* cv HHDJY) seeds were surface-sterilized by 10% NaClO, and sown on sterilized plates with MS solid culture medium (PhytoTechnology Laboratories®, Kansas, MO, USA) in a greenhouse maintained at 25 °C and with aday/night cycle of 16/8 h.

For powdery mildew infection, a strain of *G. orontii* was maintained on 3-month-old tobacco in a greenhouse. Freshly sporulating leaves of heavily infected tobacco were washed in sterile double distilled water (ddH_2_O), and the collected conidiospores were used immediately. Plants were inoculated by spraying with the inoculum suspension (about 4 × 10^4^ to 5 × 10^4^ spores ml^−1^). The 3-month-old tobacco leaves were inoculated with *G. orontii* to give approximately 20–25 spores per cm^2^ and sampled after 0, 1, 2, 12 and 24 hours post inoculation (hpi). Samples collected at 0 h were used as controls. For tissue/organ expression profiles, the root, stem and leaf were sampled on two-month-old seedlings. Flowers were taken from flowering plants and capsules were obtained during the late seed-producing period. All selected tissues and organs were stored at −80 °C.

### Total RNA isolation and quantitative real-time (qRT)-PCR expression analysis

An EasyPure Plant RNA Kit (TransGen Biotech, Beijing, China) was used to extract total RNA according to the manufacturer’s protocol. The first-strand cDNA templates were synthesized from two μg of total RNA, according to the manufacturer’s protocol (Promega, Madison, WI, USA). The reverse transcription reaction was incubated at 42 °C for 65 min in a total volume of 26 μl. The reverse transcription products were diluted five times with sterile ddH_2_O.

Specific primers were designed by Premier 5.0 according to tobacco *EDR1*-like gene sequences (Table S1). Due to the high sequence similarity between *NtEDR1-1A* and *NtEDR1-1B*, it was difficult to design specific primers for qRT-PCR to measure the expression of *NtEDR1-1A* and *NtEDR1-1B* separately*.* Therefore, one pair of primers targeting both *NtEDR1-1A* and *NtEDR1-1B* was synthesized to simultaneously check the expression levels of *NtEDR1-1A* and *NtEDR1-1B*. Each PCR reaction was mixed with 10 μl of SYBR Green (TaKaRa, Dalian, China), 6.4 μl of ddH_2_O, two μl of synthesized cDNA product and 0.8 μl of each primer (50 μM). Relative gene expression level was analyzed according to the 2^−ΔΔCt^ method (Livak & Schmittgen, 2001). Tobacco *EF-1a* gene (GenBank: D63396.1) was used as the internal reference. The real-time PCR analyses were performed using a qTOWER2.2 real-time PCR system (Analytik Jena AG, Jena, Germany), which was programmed as follows: initial 95 °C denaturation step for 3 min, followed by 40 cycles of denaturation at 95 °C for 10 s and annealing/extension at 60 °C for 1 min (Zhang et al., 2013). Every experiment was conducted with three biological replicates. All data were expressed as mean ± SEM and analyzed by Graphpad Prism 5.0 software.

## Results

### Identification of the *EDR1*-like gene family in tobacco and three other plant species

As there was no previous systematic identification and analysis of *EDR1*-like genes in plants, we identified the *EDR1*-like genes in tobacco and compared them with other model species. In total, we obtained 19, 12, 8 and 14 *EDR1*-like genes in tobacco, Arabidopsis, tomato and rice, respectively (Table S2). In addition, the basic sequence information of tobacco *EDR1*-like genes was analyzed, including the location of the *EDR1* regulatory region and the kinase region, the number of amino acids, isoelectric points, molecular weight and predicted location (Table 1).

**Table 1:** Basic information of *EDR1*-like family proteins in tobacco.

Name	Predicted location(s)	Amino acid	Isoelectronic point (pI)	Molecular weight (kDa)	Functional domains (5′–3′)	
					*EDR1* domain location	Pkinase domain location
*NtEDR1-1A*	Nucleus	986	6.11	107.85	144–343	705–956
*NtEDR1-1B*	Nucleus	1,047	6.20	114.79	144–374	735–921
*NtEDR1-2*	Nucleus	1,027	5.52	112.19	155–358	738–990
*NtEDR1-3*	Nucleus	1,027	5.64	112.42	155–358	738–990
*NtEDR1-4*	Nucleus	1,032	5.30	112.83	146–350	754–1,006
*NtEDR1-5*	Nucleus	895	5.73	99.09	138–216, 213–313	625–877
*NtEDR1-6*	Chloroplast, Nucleus	780	–	–	1–83, 92–219	501–762
*NtEDR1-7*	Nucleus	846	5.74	93.33	196–423	570–824
*NtEDR1-8*	Nucleus	846	5.74	93.33	196–423	570–824
*NtEDR1-9*	Nucleus	848	5.68	93.40	200–407	572–826
*NtEDR1-10*	Nucleus	776	5.38	85.15	146–353	525–774
*NtEDR1-11*	Cell membrane	790	5.83	89.26	55–254	534–783
*NtEDR1-12*	Nucleus	891	6.25	99.48	290–497	683–890
*NtEDR1-13*	Nucleus	1,240	7.56	138.74	482–681	984–1,232
*NtEDR1-14*	Cell membrane	814	6.04	91.61	56–255	558–806
*NtEDR1-15*	Nucleus	743	5.08	81.32	146–53	525–633, 619–741
*NtEDR1-16*	Nucleus	876	6.16	98.03	288–495	681–875
*NtEDR1-17*	Cell membrane Nucleus	648	6.36	73.67	55–166	392–641
*NtEDR1-18*	Nucleus	892	5.02	97.15	146–350	747–858

The *NtEDR1* regulatory region was located at the N-terminal, whereas the kinase region was located at the C-terminal of the predicted protein sequence. The C-terminal kinase region shared a sequence identity of 61.48%, and the N-terminal regulatory region had a relatively low identity of 40.52% for the predicted protein sequences of tobacco *EDR1*-like genes (Fig. S1). The number of amino acids ranged from 648 (*NtEDR1-17*) to 1,240 (*NtEDR1-13*). The predicted molecular masses were in the range of 73.67–138.74 kDa and the isoelectric points were 5.02–6.36. Protein subcellular localization prediction is a key step in understanding the protein function and its protein network interaction pattern (Chou & Shen, 2007). In Arabidopsis, at least a fraction of the *EDR1* protein has been shown to localize in the nucleus (Christiansen et al., 2011). Our results showed 15 out of 19 tobacco *EDR1*-like proteins were predicted to be located in the nucleus (Table 1), but *NtEDR1-11* and *NtEDR1-14* were predicted to be located in the cell membrane. The other two tobacco *EDR1*-like members, *NtEDR1-6* and *NtEDR1-17*, were shown to be located in the chloroplast or nucleus, and cell membrane or nucleus, respectively. The NLS predictions for most genes were consistent with those of subcellular localization, which showed that 13 *NtEDR1*-like proteins had NLS sequences (Table 1; Table S3). However, there were differences for another six *NtEDR1*-like proteins (Table 1; Table S3). This might be due to the different algorithms used for the two tools.

### Phylogenetic and analysis of *EDR1*-like genes

**Figure 1:**
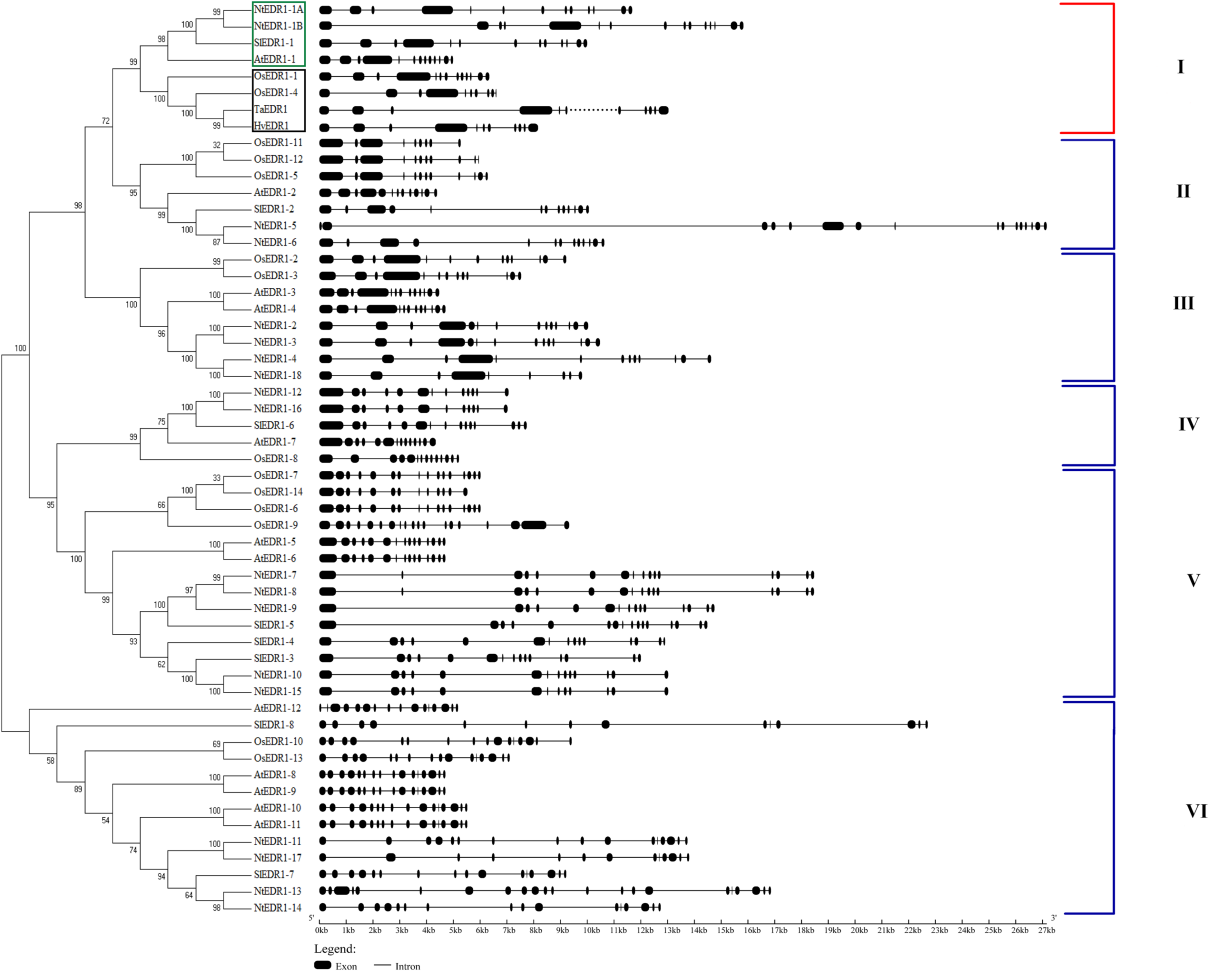
Phylogenetic relationships and gene structure of *EDR1*-like gene families from tobacco, Arabidopsis, rice and tomato. *NtEDR1-1A* and *NtEDR1-1B*, along with *EDR1* homologs involved in negative regulation of resistance against powdery mildew in Arabidopsis (*AtEDR1-1*) and wheat (*TaEDR1*), were grouped into clade I, in which dicotyledon plants are shown with green box, and black box represents monocotyledons. The dotted line represents that there is gap in the available genome sequence of that gene. The GenBank accession number of the barley *EDR1* protein sequence is AAG31142.1 (*HvEDR1*). The genome sequences of barley and wheat were downloaded from the genome databases (ftp://ftp.ensemblgenomes.org/pub/).

**Figure 2:**
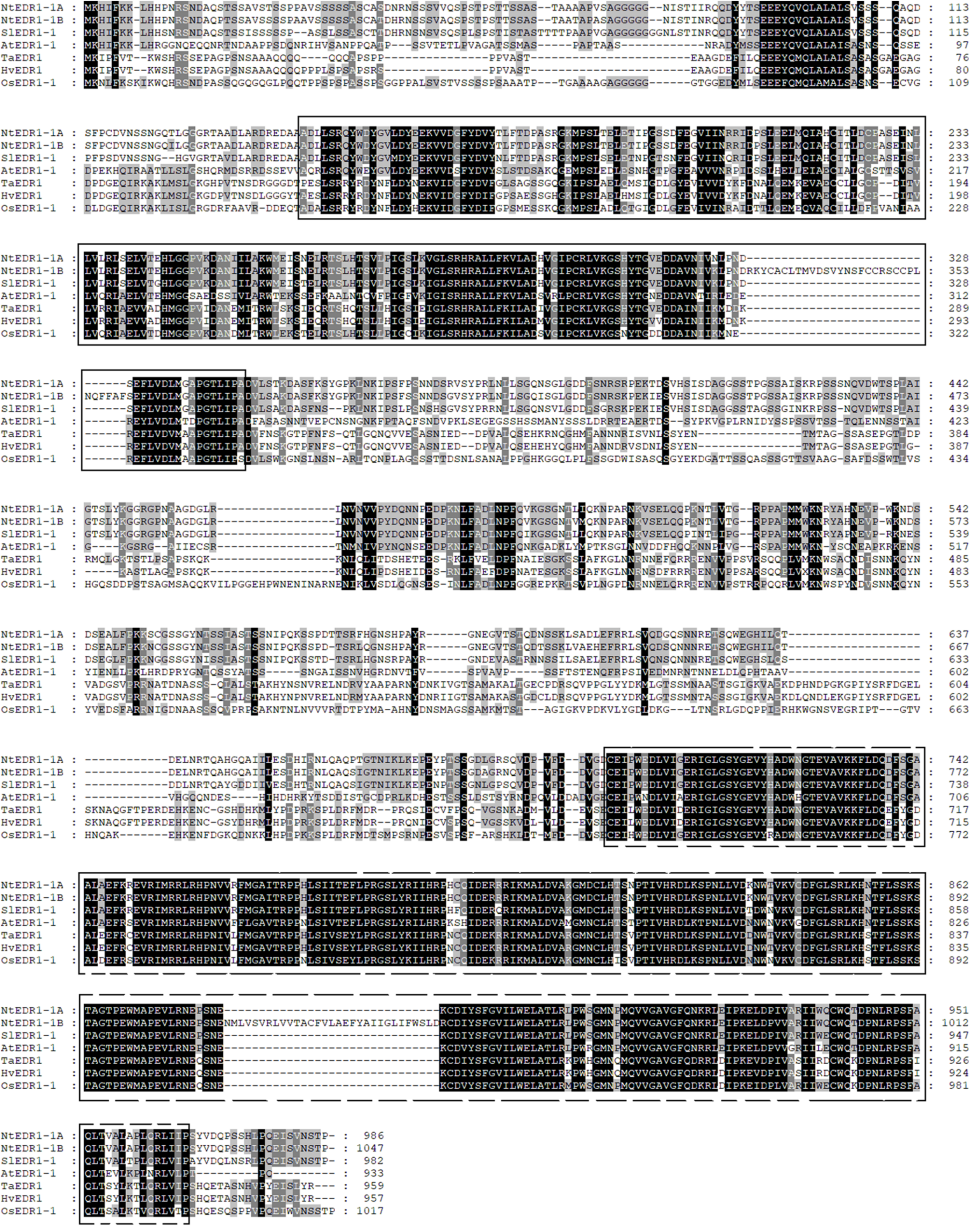
Protein sequence alignment of *EDR1-1* from tobacco, Arabidopsis, rice and tomato. Positions of *EDR1* N-terminal regulatory domains are indicated by a black-lined box; and a dashed box indicates kinase domains.

Reports have shown that *EDR1* homologs play a negative role in the defense response against powdery mildew in Arabidopsis (Frye & Innes, 1998) and wheat (Zhang et al., 2017). To reveal the evolutionary relationships among different plant species, a phylogenetic tree including *EDR1*-like gene members in tobacco, Arabidopsis, wheat and rice was constructed using software MEGA 7.0 with NJ method (Fig. 1). The results divided the *EDR1*-like genes into six clades and the monocot and eudicot *EDR1*-like genes were separated into two sub-families within each clade (Fig. 1). *NtEDR1-1A* and*NtEDR1-1B*, along with *EDR1* homologs involved in negative regulation of resistance against powdery mildew in Arabidopsis (*AtEDR1-1*) and wheat (*TaEDR1*), were grouped into clade I. Thus, *NtEDR1-1A* and *NtEDR1-1B* should be the first choice for further study of any negative roles in tobacco defense against powdery mildew. The exon–intron organizations of the *EDR1*-like genes were analyzed using online software GSDS (Fig. 1). The exon number of tobacco *EDR1*-like genes varied from nine (*NtEDR1-18*) to 22 (*NtEDR1-13*). Interestingly, *NtEDR1-1A*, *AtEDR1-1*, *SlEDR1-1* and *OsEDR1-1* had 13 exons and were all grouped into clade I in the phylogenetic tree. The similarities of sequences in clade I were further analyzed by multiple sequence alignment. The results showed that both the predicted C-terminal kinase region and N-terminal regulatory region of sequences in clade I had high identities (82.29% and 66.89%, respectively) (Fig. 2).

### Conserved motif analysis and structure prediction of tobacco*EDR1*-like protein

**Figure 3:**
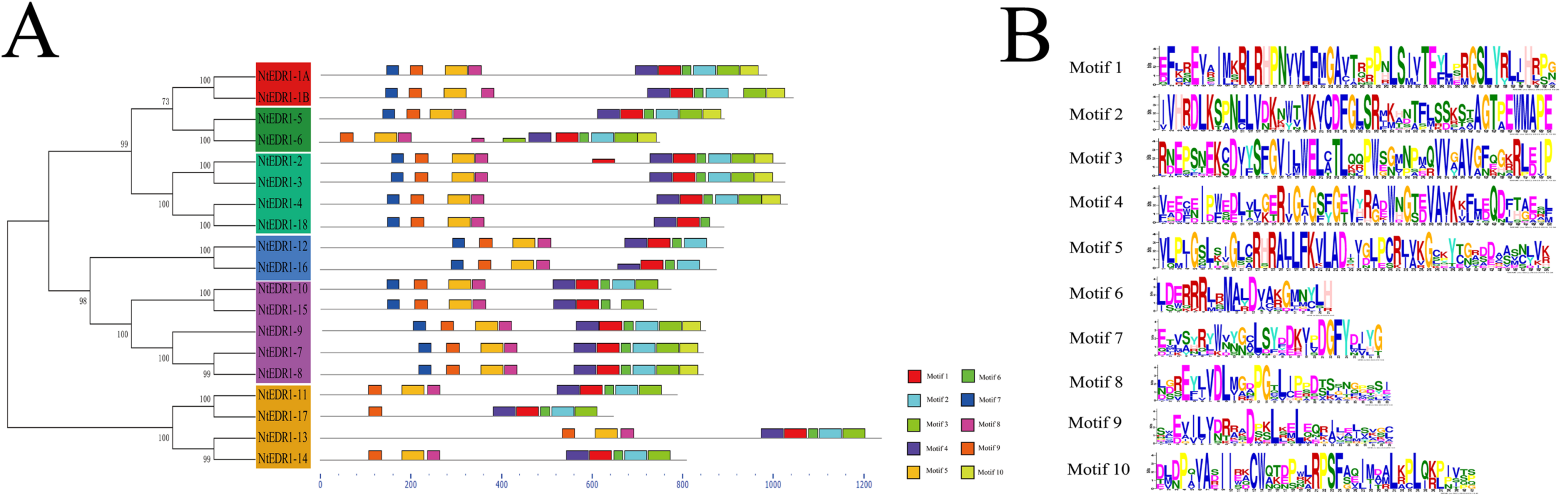
Distribution of conserved motifs in the tobacco *EDR1*-like family members. (A) Conserved motifs in *NtEDR1*-like proteins were analyzed by MEME. Ten different motifs are represented by different colored boxes, and their sizes could be estimated by the scale at the bottom. Details of motif were shown in Table S4. (B) The heights of each box represent the specific amino acid conservation in each motif.

To further analyze the conserved features of the *NtEDR1*-like family proteins, the motif of the tobacco *EDR1*-like proteins were predicted by MEME web server. A total of 10 motifs were identified in the *NtEDR1*-like proteins (Fig. 3; Table S4). According to the prediction of SMART, motifs 1–4 and 10 were located within the kinase domain, while motif 5 was in the *EDR1* domain. The results showed motifs 1, 4, 6 and 9 were necessary for all the tobacco *EDR1*-like members, but motifs 2, 3, 5, 7 and 8 were alternative components (Fig. 3A). The motif logo is shown in Fig. 3B, and the lengths and predicted motif models of each motif are shown in Table S4.

**Figure 4:**
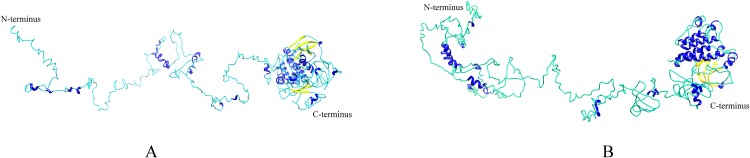
The predicted 3D structures of *NtEDR1-1* and *AtEDR1-1*. (A) A 3D structure pattern of *NtEDR1-1A*. (B) A 3D structure pattern of *AtEDR1-1*. Blue represents α-helices, yellow represents β-strands and cyan represents random coils. Structural images were generated with Chimera 1.2.

Homologous proteins with similar function often have a similar structure. *AtEDR1-1* has been experimentally shown to be required for *G. cichoracearum* susceptibility (Frye & Innes, 1998), so we used *AtEDR1-1* as a reference model to analyze *NtEDR1*-like proteins. The 3D structures of tobacco *EDR1*-like proteins and *AtEDR1-1* were constructed by using I-TASSER web site. The results showed that the predicted 3D model of *NtEDR1-1A* was similar to that of *AtEDR1-1*, while the 3D models of the rest tobacco *NtEDR1*-like proteins were somewhat different (Fig. 4; Fig. S2).

### Expression analysis of *EDR1*-like genes in tobacco

**Figure 5:**
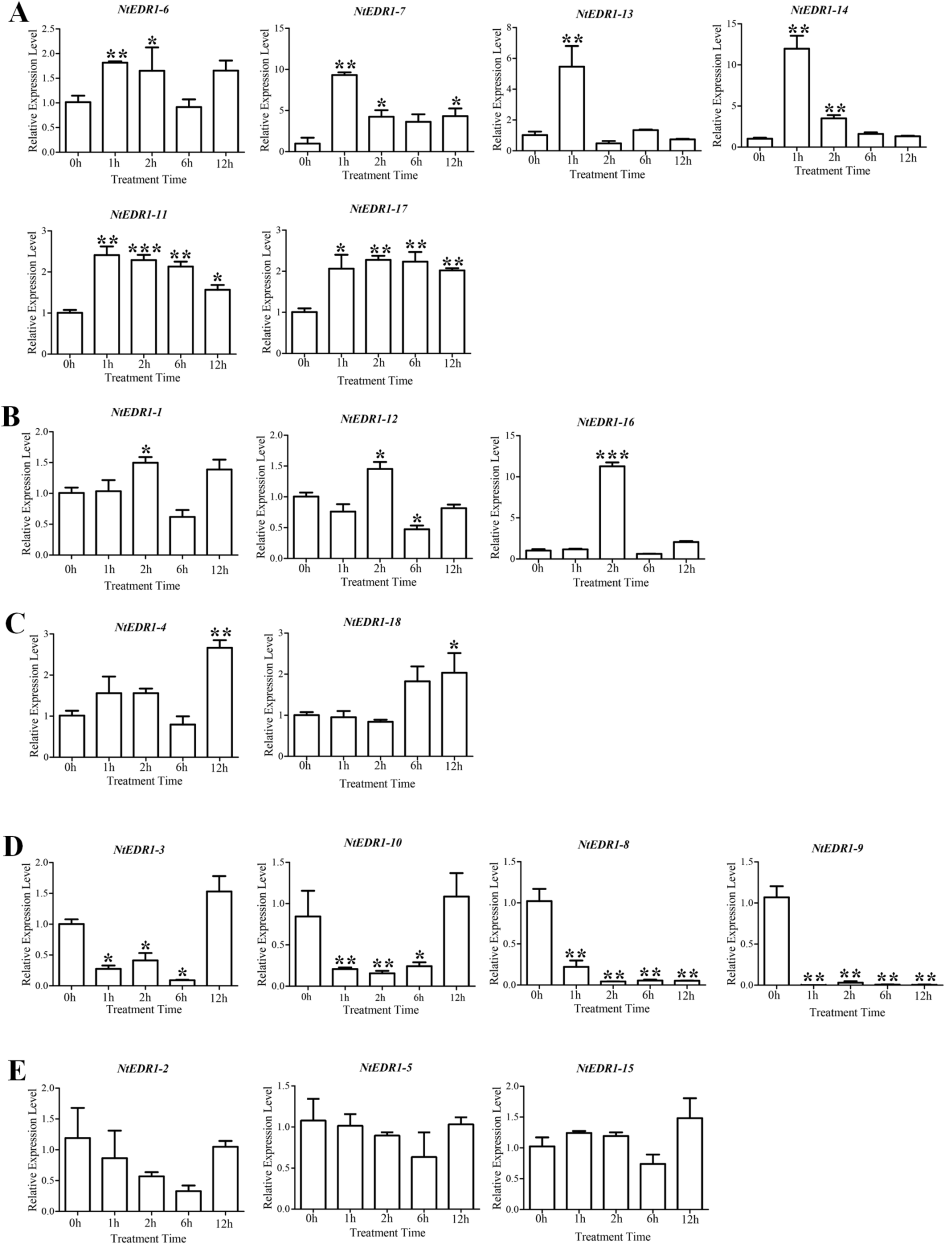
Expression profiles of *NtEDR1*-like genes in tobacco leaves in response to *Golovinomyces orontii* infection. Leaves were inoculated with *G. orontii* to give approximately 20–25 spores per cm^2^ and sampled after 0, 1, 2, 12 and 24 h (0 h as control). The expression levels of the genes were significantly increased at 1 h (A), increased at 2 h (B), increased at 12 h (C), decreased (D), and not signifcantly altered (E). ***, ** and * indicate significant differences in comparison with the control at *P* < 0.001, *P* < 0.01 and *P* < 0.05, respectively.

The qRT-PCR was employed to determine the expression of tobacco *EDR1*-like genes after plants were infected with *G. orontii* (Fig. 5). *NtEDR1-1* was used to represent both *NtEDR1-1A* and *NtEDR1-1B* where necessary to describe the transcript abundance of *NtEDR1-1A* and *NtEDR1-1B.* The result showed that *NtEDR1-1*, *NtEDR1-12* and *NtEDR1-16* had similar expression patterns (Fig. 5B). These genes were not immediately up-regulated but began to increase after 2 hpi. *NtEDR1-17* was up-regulated at 1 hpi, and maintained a high level of expression at 2 hpi. *NtEDR1-3* and *NtEDR1-10* weredown-regulated in the beginning of infection, but up-regulated at 12 hpi. *NtEDR1-8* and *NtEDR1-9* were significantly down-regulated by *G. orontii* infection. *NtEDR1-2*,*NtEDR1-5* and *NtEDR1-15* showed no obvious changes at all time points tested.*NtEDR1-4* and *NtEDR1-18* were up-regulated at 12 hpi. *NtEDR1-6* and *NtEDR1-11* were up-regulated at 1 hpi; subsequently, expression of *NtEDR1-6* decreased, and *NtEDR1-11* maintained high expression after 2 hpi. *NtEDR1-7*, *NtEDR1-13* and *NtEDR1-14* were immediately up-regulated with *G. orontii* infection at 1 hpi, but their expression declined to a normal level at 6 hpi. These results indicated that different *EDR1*-like genes showed different expression patterns after *G. orontii* challenge. The expression patterns of *NtEDR1-1*, *NtEDR1-12* and *NtEDR1-16* were similar after tobacco was challenged by *G. orontii*.

Analysis of transcriptional level of *NtEDR1*-like genes in different tissues/organ samples provides clues on their biological functions. The tissue/organ expression patterns of tobacco *NtEDR1*-like genes were also investigated using qRT-PCR (Fig. S3). Some *NtEDR1*-like genes showed organ-specific expression patterns in the organ sample investigated. For example, *NtEDR1-13* and *NtEDR1-16* were expressed highly only in roots and leaves, respectively (Fig. S3A). The expression of *NtEDR1-1*, *NtEDR1-3*, *NtEDR1-7*, *NtEDR1-8*, *NtEDR1-10* and *NtEDR1-15* were absent in flowers; and *NtEDR1-4*, *NtEDR1-9*, *NtEDR1-11*, *NtEDR1-14*, *NtEDR1-17* and *NtEDR1-18* did not show any expression in capsules. *NtEDR1-2*, *NtEDR1-5*, *NtEDR1-6* and *NtEDR1-12* had some transcripts in all organ samples tested (Figs. S3B–S3D).

## Discussion

The *EDR1* genes were first reported to be required for *G. cichoracearum* susceptibility in Arabidopsis (Frye & Innes, 1998), and are also involved in regulation of multiple physiological activities (Kim et al., 2003). For example, they play a role in the salicylic acid pathway under disease stress, and have a function in the ethylene pathway under drought stress (Rodriguez, Petersen & Mundy, 2010; Tang, Christiansen & Innes, 2005; Tang & Innes, 2002). Moreover, *EDR1* could be recruited by *EDR4* to the fungal penetration site via physical interaction (Wu et al., 2015). *EDR4* has been shown to encode an unknown protein that might function in the same pathway with *EDR1* to regulate powdery mildew resistance and cell death (Wu et al., 2015). Therefore, it is necessary to further explore the physiological function of *EDR1* members. *EDR1*s have been found in several plants; however, there were no previous reports on *EDR1*-like genes in tobacco. In this study, we identified 19 *EDR1*-like genes in tobacco and compared these with the *EDR1*-like gene family from Arabidopsis, tomato, and rice. Tissue/organ and *G. orontii*-induced expression patterns of tobacco *EDR1*-like genes were further analyzed.

Phylogenetic analysis divided the *EDR1*-like genes into six clades. *NtEDR1-1A* and *NtEDR1-1B—*along with the reported *EDR1*s, *AtEDR1-1* and *TaEDR1*, negatively functioning in resistance to powdery mildew—clustered in clade I. The results of 3D model prediction showed that *NtEDR1-1A* was most similar to that of *AtEDR1-1* among all the *NtEDR1*-like genes tested (Fig. 4; Fig. S2). Multi-sequence alignment analysis revealed that genes in clade I showed high similarities, with 82.29% identity of the C-terminal kinase region and 66.89% identity of the N-terminal regulatory region. This suggested that the *EDR1* regulatory region and kinase domain were highly conserved in clade I. The pathway of *EDR1* homologs is likely conserved between monocots and eudicots (i.e., maize, rice and tomato) (Frye, Tang & Innes, 2001). The *EDR1*-like members in each clade were divided into monocot and dicot sub-families. Multi-sequence alignment analysis of tobacco *EDR1*-like proteins showed the C-terminal kinase region had relatively high identity (61.48%), but the N-terminal regulatory region had relatively low identity (40.52%). As *EDR1* function is dependent upon the N-terminal regulatory region (Tang, Christiansen & Innes, 2005), the lower identity among the N-terminal regulatory regions of tobacco *EDR1*-like genes may indicate that they have different functions. Motif distribution analysis showed that different *NtEDR1*-like members had different numbers and types of motifs (Fig. 3A). Genomic structure analysis also showed that the exon–intron structure of *EDR1*-like genes greatly differed among species.

Quantitative expressions of tobacco *EDR1*-like genes following infection by *G. orontii* were analyzed. The results showed 15 tobacco *EDR1*-like genes were *G. orontii*-susceptible (Figs. 5A–5D): *NtEDR1-1*, *NtEDR1-12* and *NtEDR1-16* were up-regulated at 2 hpi; *NtEDR1-6*, *NtEDR1-7*, *NtEDR1-11*, *NtEDR1-13*, *NtEDR1-14* and *NtEDR1-17* were immediately up-regulated after *G. orontii* infection; *NtEDR1-4* and *NtEDR1-18* were up-regulated at 12 hpi; and *NtEDR1-3*, *NtEDR1-8*, *NtEDR1-9* and *NtEDR1-10* were down-regulated at 1 hpi. Knockout of three homologs of *TaEDR1* in wheat did not produce complete resistance (Zhang et al., 2017), and separately silencing the tomato homologs of *EDR1* (Solyc01 g097980 and Solyc06 g068980) did not result in significant resistance to *O. neolycopersici* (Gao et al., 2015). It is necessary to silence multiple *EDR1* genes to obtain plants with enhanced resistance (Gao et al., 2015). This indicated that multiple *EDR1* members may be commonly involved in the negative regulation of resistance against powdery mildew in tobacco and many other plants. The other three *NtEDR1*-like genes (*NtEDR1-2*, *NtEDR1-5* and *NtEDR1-15*) did not show significantup- or down-regulation after plant infection by *G. orontii* (Fig. 5E). Therefore, not all tobacco *EDR*1-like genes responded to *G. orontii* infection. Plant basic defenses require the MKK4/MKK5-MPK3/MPK6 kinase cascade, and *EDR1* physically associates with MKK4/MKK5 and negatively regulates the MAPK cascade to fine-tune plant innate immunity (Zhao et al., 2014). Several reports have shown that mutations of *EDR1* could confer plant disease resistance in Arabidopsis, rice and wheat (Frye & Innes, 1998;Shen et al., 2011; Zhang et al., 2017). Therefore, it might be common that presence of *EDR1* may inhibit the plant primary immunity and benefit invasion by powdery mildew.

Tissue/organ expression analysis of tobacco *EDR1*-like genes was carried out usingqRT-PCR. The results showed that different *EDR1*-like genes had different expression patterns. *NtEDR1-2*, *NtEDR1-3*, *NtEDR1-4*, *NtEDR1-9* and *NtEDR1-10* had higher expression levels in roots. *NtEDR1-1*, *NtEDR1-5*, *NtEDR1-7*, *NtEDR1-8*, *NtEDR1-12* and *NtEDR1-15* were predominantly expressed in capsules. *NtEDR1-11* and *NtEDR1-17* were highly expressed in flowers. Interestingly, two *NtEDR1*-like genes, *NtEDR1-13* and *NtEDR1-16*, were exclusively expressed in roots and leaves, respectively. In addition to functions as negative regulators of plant defense, *EDR1*-like genes have also been shown to have roles in other physiological processes. For example, *OsEDR1-1* (Os3 g06410) had a high expression level during maturation of the panicle before heading, after heading and at maturity (pollination stage) in rice, and was further suggested to play a role in plant growth and development, and in maturity of panicles (Kim et al., 2003). In our study, *NtEDR1-11* and *NtEDR1-17* were mainly expressed in flowers, whereas *NtEDR1-5*, *NtEDR1-7*,*NtEDR1-8*, *NtEDR1-12* and *NtEDR1-15* were highly expressed in capsules. Thus, some tobacco *EDR1*-like genes may also have a physiological function during the plant reproductive period.

## Conclusions

In this research, 19 *EDR1*-like genes were identified in a genome-wide analysis in *N. tabacum*. Through multiple sequences alignment, phylogenetic analysis, gene structure analysis and comparative analysis of predicted 3D structures, *NtEDR1-1A* was shown to have the highest similarity to *AtEDR1-1*, which has been reported to be responsive to powdery mildew. Expression profiles revealed that *NtEDR1-1* (*NtEDR1-1A* and *NtEDR1-1B*) was not the only tobacco *EDR1*-like member responsive to *G. orontii*, but not all tobacco *EDR1*-like genes were responsive to *G. orontii*. Tissue/organ expression profiles showed that different *EDR1*-like members had different expression patterns, but some *NtEDR1*-like genes showed tissue-specific expression patterns. Our results provide foundational information on the *EDR1*-like gene family in tobacco species, and promise to promote future research on the functions of *EDR1*-like genes in plants.

## Supplemental Information

10.7717/peerj.5244/supp-1 Supplemental Information 1 Multiple sequences alignment of the protein sequences of tobacco *EDR1-like* genes.

10.7717/peerj.5244/supp-2 Supplemental Information 2 The predicted 3D structures of tobacco *EDR1-like* genes.

10.7717/peerj.5244/supp-3 Supplemental Information 3 Expression profile of tobacco *EDR1-like* genes in different tissue/organ samples. Root-Y, Stem-Y and Leaf-Y indicate young root, young stem and young leaf, respectively. Flower-F indicates open flowers. Capsule-seed indicates the capsules that were obtained during the late seed-breeding period. The genes have clear tissue-specific expression patterns (A); The genes expressed in all organ samples tested (B); The expression of genes were absent in flowers (C); and the genes did not show any expression in capsules (D).

10.7717/peerj.5244/supp-4 Supplemental Information 4 The protein sequences of Arabidopsis *EDR1-like* genes.

10.7717/peerj.5244/supp-5 Supplemental Information 5 The protein sequences of tobacco *EDR1-like* genes.

10.7717/peerj.5244/supp-6 Supplemental Information 6 The protein sequences of tomato *EDR1-like* genes.

10.7717/peerj.5244/supp-7 Supplemental Information 7 The protein sequences of rice *EDR1-like* genes.

10.7717/peerj.5244/supp-8 Supplemental Information 8 The primer sequences used in qRT-PCR for detection the expression patterns of *NtEDR1-like* genes.

10.7717/peerj.5244/supp-9 Supplemental Information 9 Summary of the *EDR1-like* genes in different plant species. The protein sequences of *EDR1-like* genes in each species is presented in Supplementary Materials (Arabidopsis in File S1; tobacco in File S2; tomato in File S3; and rice in File S4).

10.7717/peerj.5244/supp-10 Supplemental Information 10 Prediction of NLS sequences and the existence of transmembrane helices of the tobacco EDR1-like proteins. “√” represents protein has transmembrane helices.

10.7717/peerj.5244/supp-11 Supplemental Information 11 Putative motifs conserved in the amino acid sequences of the tobacco EDR1-like proteins.

## Additional Information and Declarations

### Data Availability

The following information was supplied regarding data availability: The raw data are provided in the Supplemental Files.
