# Supplementary figures and images for: Identification and expression analysis of EDR1-like genes in tobacco (Nicotiana tabacum) in response to Golovinomyces orontii
Source: PeerJ. 2018 Jul 10;6:e5244. doi: 10.7717/peerj.5244 (PMC6044316; doi:10.7717/peerj.5244)

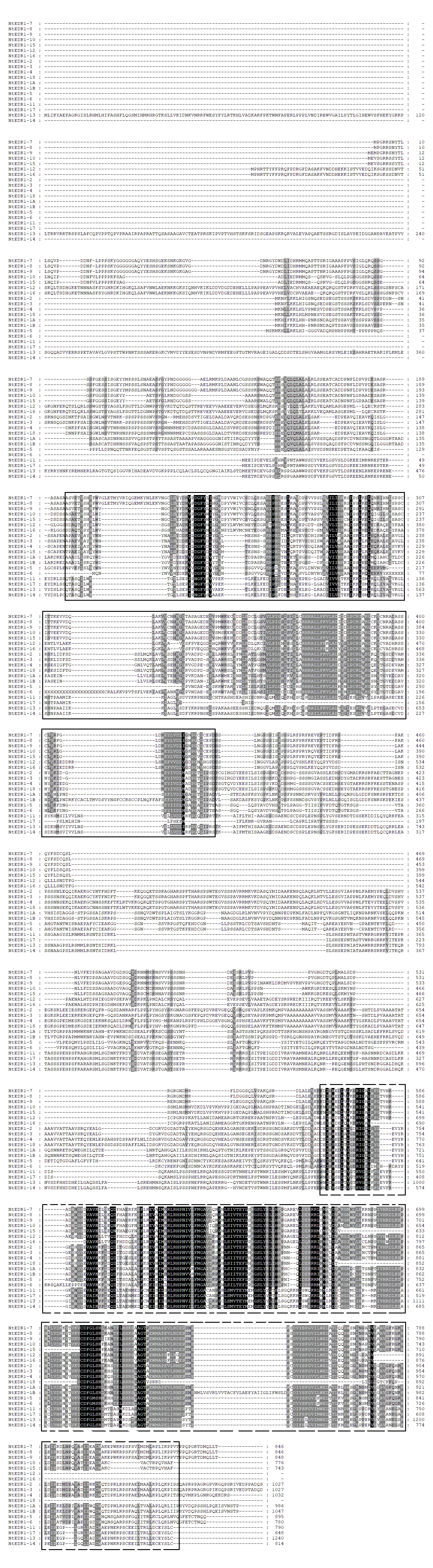

Supplement: Supplemental Information 1 [file peerj-06-5244-s001.png]

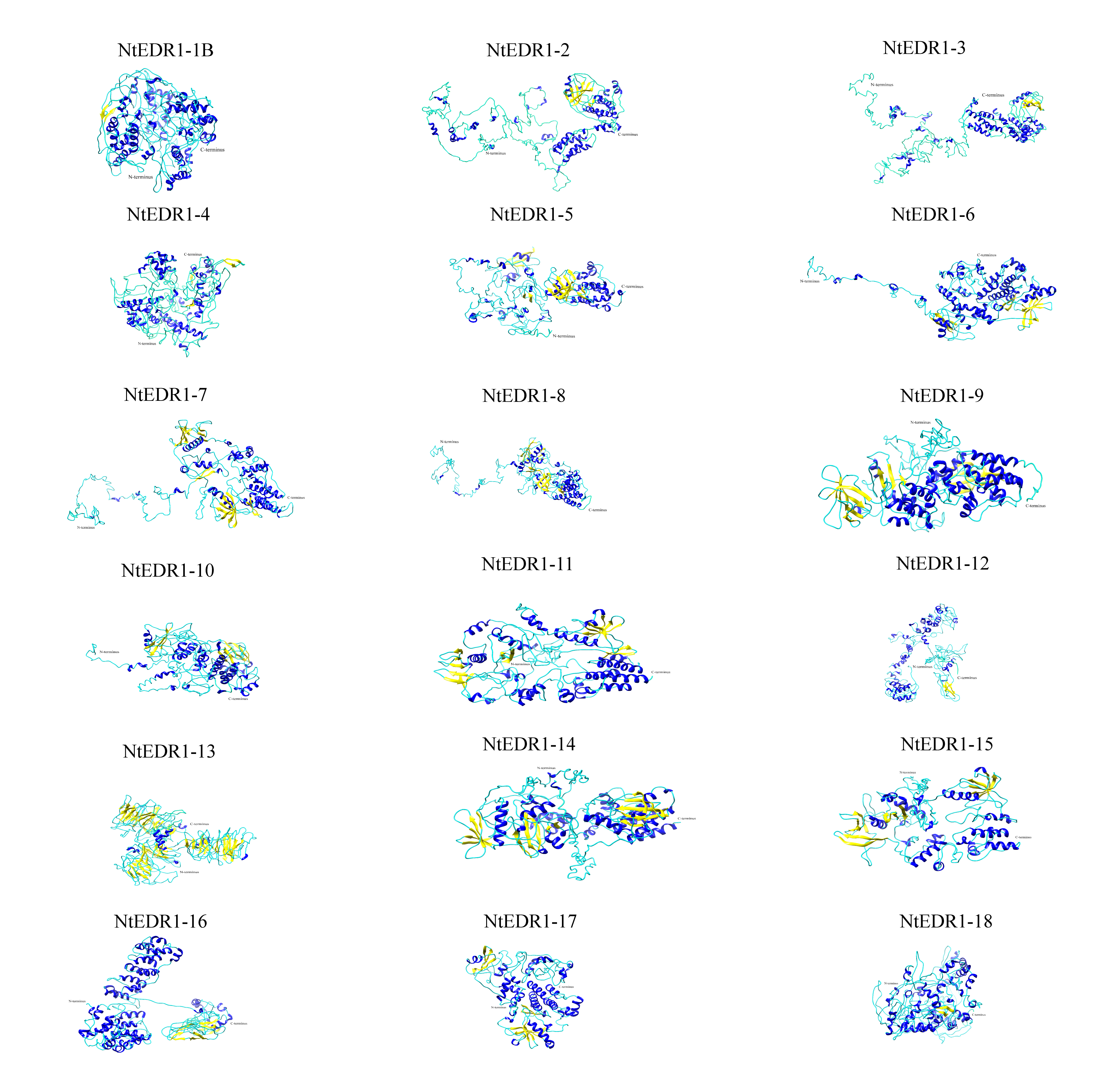

Supplement: Supplemental Information 2 [file peerj-06-5244-s002.png]

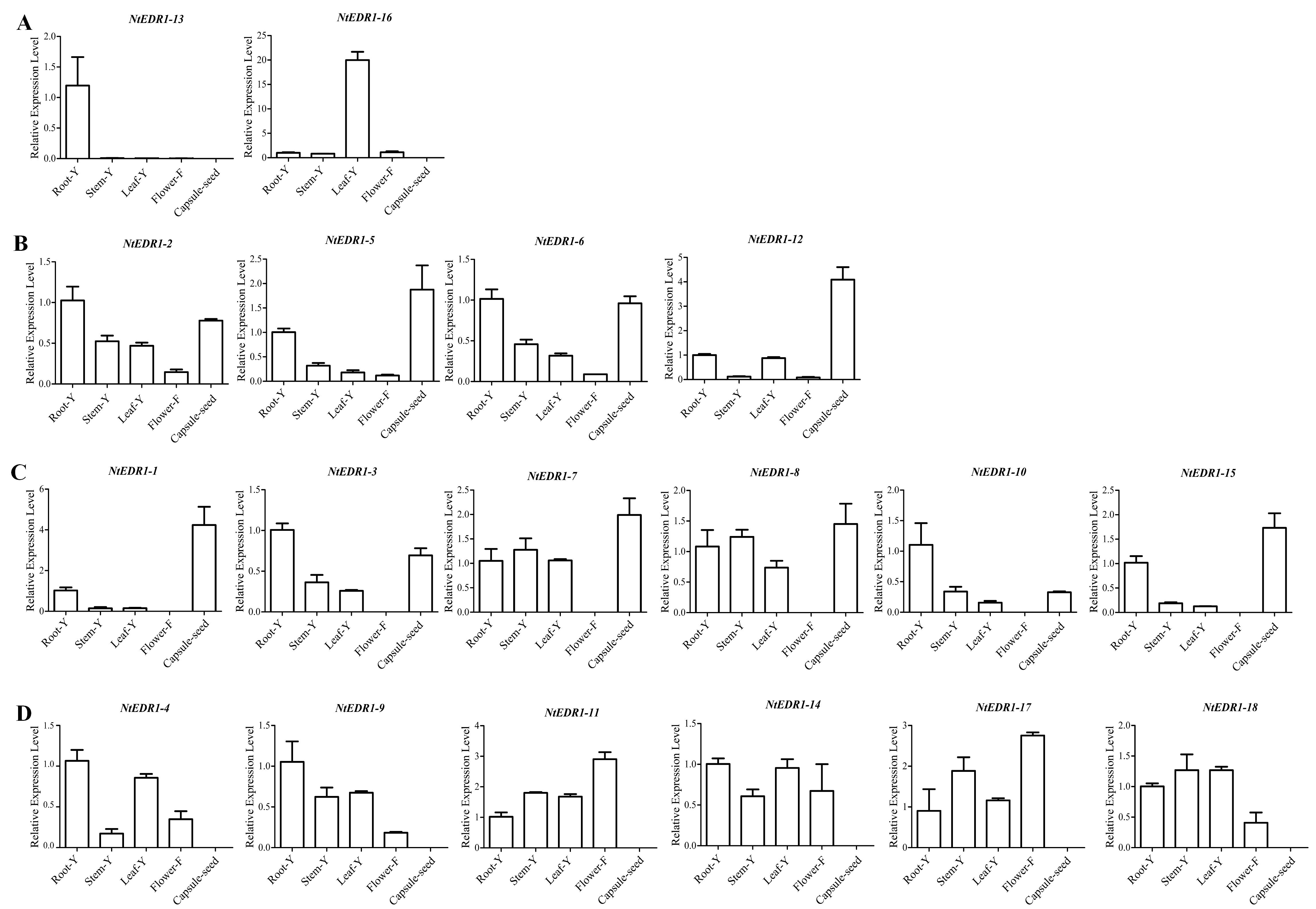

Supplement: Supplemental Information 3 — Root-Y, Stem-Y and Leaf-Y indicate young root, young stem and young leaf, respectively. Flower-F indicates open flowers. Capsule-seed indicates the capsules that were obtained during the late seed-breeding period. The genes have clear tissue-specific expression patterns (A); The genes expressed in all organ samples tested (B); The expression of genes were absent in flowers (C); and the genes did not show any expression in capsules (D). [file peerj-06-5244-s003.png]
