# Supplementary material for: Identification and expression analysis of EDR1-like genes in tobacco (Nicotiana tabacum) in response to Golovinomyces orontii
Source: PeerJ. 2018 Jul 10;6:e5244. doi: 10.7717/peerj.5244 (PMC6044316; doi:10.7717/peerj.5244)
Supplement: Supplemental Information 4 [file peerj-06-5244-s004.docx]

>AtEDR1-1

MKHIFKKLHRGGNQEQQNRTNDAAPPSDQNRIHVSANPPQATPSSVTETLPVAGATSSMASPAPTAASNRADYMSSEEEYQVQLALAISASNSQSSEDPEKHQIRAATLLSLGSHQRMDSRRDSSEVVAQRLSRQYWEYGVLDYEEKVVDSFYDVYSLSTDSAKQGEMPSLEDLESNHGTPGFEAVVVNRPIDSSLHELLEIAECIALGCSTTSVSVLVQRLAELVTEHMGGSAEDSSIVLARWTEKSSEFKAALNTCVFPIGFVKIGISRHRALLFKVLADSVRLPCRLVKGSHYTGNEDDAVNTIRLEDEREYLVDLMTDPGTLIPADFASASNNTVEPCNSNGNKFPTAQFSNDVPKLSEGEGSSHSSMANYSSSLDRRTEAERTDSSYPKVGPLRNIDYSSPSSVTSSTQLENNSSTAIGKGSRGAIIECSRTNMNIVPYNQNSEEDPKNLFADLNPFQNKGADKLYMPTKSGLNNVDDFHQQKNNPLVGRSPAPMMWKNYSCNEAPKRKENSYIENLLPKLHRDPRYGNTQSSYATSSSNGAISSNVHGRDNVTFVSPVAVPSSFTSTENQFRPSIVEDMNRNTNNELDLQPHTAAVVHGQQNDESHIHDHRKYTSDDISTGCDPRLKDHESTSSSLDSTSYRNDPQVLDDADVGECEIPWNDLVIAERIGLGSYGEVYHADWHGTEVAVKKFLDQDFSGAALAEFRSEVRIMRRLRHPNVVFFLGAVTRPPNLSIVTEFLPRGSLYRILHRPKSHIDERRRIKMALDVAMGMNCLHTSTPTIVHRDLKTPNLLVDNNWNVKVGDFGLSRLKHNTFLSSKSTAGTPEWMAPEVLRNEPSNEKCDVYSFGVILWELATLRLPWRGMNPMQVVGAVGFQNRRLEIPKELDPVVGRIILECWQTDPNLRPSFAQLTEVLKPLNRLVLPTPQ

>AtEDR1-2

MSKMKHLLRKLHIGGSSGVGGGFADHHRLDDSTRPMIDPSPILSTSPSPASTSSVSSSGFGNASTTMPRLDTFEPVGRDLTAVDGVDFNLMEEEYQVQLAMAISVSDPDPRENADTAQLDAAKRISLGVSAPVTDADSAVDFLSLRYWGHKVINYDQKVRDGFYDVYGITSNSLSQGKMPLLVDLQAISISDNVDYEVILVNRLIDPELQELERRVFALASECPDFAPGQVSSDLTQKIANIVVEQMGGPVENADEALRRWMLRSYELRNSLNTTILPLGRVNVGLARHRALLFKVLADRINLPCMLVKGSYYTGTDDGAVNLIKLDDKSEYIIDLMGAPGALIPSEVPSSFLPVSCTDTRVFPENLDSLQHSSPVLEKEIETPAFSVSKEADSRSGMVANFFTGNQEENSDRCAVEKHQTERFEHDFGKLMHSQQISGENMPPFSGKPTCAQKVKVKNVSKYVISAAKNPEFAQKLHAVLLESGASPPPDLFMDINPHNLRGKNLLQELRQESSNSMVSGIPCYPEKVAEQLRESERNPTAESYQQSVEVDLSMKRNFDLDNTGKASSSENMEVGTADGESAVCDSHDQGINPLLGEAAKWEIMWEDLQIGERIGIGSYGEVYRAEWNGTEVAVKKFLDQDFSGDALTQFKSEIEIMLRLRHPNVVLFMGAVTRPPNFSILTEFLPRGSLYRLLHRPNHQLDEKRRMRMALDVAKGMNYLHTSHPTVVHRDLKSPNLLVDKNWVVKVCDFGLSRMKHHTYLSSKSTAGTPEWMAPEVLRNEPANEKCDVYSFGVILWELATSRVPWKGLNPMQVVGAVGFQNRRLEIPDDIDLTVAQIIRECWQTEPHLRPSFTQLMQSLKRLQGLNISNRANTSESLM

>AtEDR1-3

MKVKEETLKNLGDGVVLRPVDHCSSIWSMKMNMKNFLKKLHISPNQSDEAEGSISTTKSNHHKSIDVSSSSSPRSHHSNSPEIKPFSGLSNWLSSVGHRKIPSPPNSFNAKNRAATVDDTVVVNGSEHVDLGSKDPAVEEENQIQLALELSAREDPEATQIEAIKQFSLGSCAPENSPAELIAYRYWNYNCLGYDDKILDGFYDLYGVLNASSAERIPPLLDLQGTPVSDGVTWEAVLVNRSGDSNLLRLEQMALDIAAKSRSVSSSGFVNSELVRKLAILVGDYMGGPVVHPESMLRAWRSLSYSLKATLGSMVLPLGSLTIGLARHRALLFKVLCDSVGVPCRIVKGQQYTGSEDVAMNFIKADDGREYIVDLMGDPGTLIPADAAGLQIDYDESAYSASPGDNDSIHVASSSNGIESSYEENTEFRTGEHRSSTKSSGERNQSGGGGDLIVHPNISREDVKNQKKVEKAPFQNLSSRPIHSFTHMRSPSWTEGVSSPAAQRMKVKDVSQYMIDAAKENPRLAQKLHDVLLESGVVAPPNLFSEVYPQQLEATVESKNSTEAKKERGKDLETTQEGRHQNGFGPVRFLPPLPRVQSKTNAHDQRDNGKVVSQSDSSHSEASSTEYARTVPAAVAAAAVVASSMVAAAAAKSANSDSSPIELPAAAAATATAAAVVATAAAVSRQLELGSNSDGDDGSGGHEPQGSGDSNHGPNSGGERISDKSIGNESSKSDCDDVSDCEILWEEITVGERIGLGSYGEVYRGDWHGTEVAVKKFLDQDLTGEALEEFRSEVRIMKKLRHPNIVLFMGAVTRPPNLSIVTEFLPRGSLYRLIHRPNNQLDERRRLRMALDAARGMNYLHSCNPMIVHRDLKSPNLLVDKNWVVKVCDFGLSRMKHSTYLSSKSTAGTAEWMAPEVLRNEPADEKCDVYSYGVILWELFTLQQPWGKMNPMQVVGAVGFQHRRLDIPDFVDPAIADLISKCWQTDSKLRPSFAEIMASLKRLQKPVTGSNIPRPVPSSSSLPTEHEQKD

>AtEDR1-4

MKMNMKKFLKKLRITPNQRDDGEGSVSNRSNKSSDAEPSPSDSLRSQDNSEFKPFLGLSNWLSSVTHRKSPSSSNATNSKEDDTTMEHGGPVGSESGMQGLGSSSNSKDPEVEEEYQIQLALELSAREDPEAAQIEAMKQFSLGSRPSAPENTPAELMAYRYWNYNCLGYDDKIVDGFYDLCGVMNESSLKRIPPLVDLQGTLVSDGVTWDAVLVNSSKDSNLLRLEQMALDIAAKSKSASSSGFVNSELVRQLAVLVADYMGGPVLDPDSTLRAWWSLSYSLKATLRSMVLPLGSLTIGLARHRALLFKVLCDSVGVPCRIVKGQQYTGSDDVAMNSIKTDDGREYIVDLMGDPGTLIPADAAGLQMDFDDSVYSASPRDVDSSHVASSSSGVESSIEEHTESWSAEHRSRTKGSREENQSAGGGDLMIPNIREAVGSQKAPVQHLSSKPTHSFTHARSPSWTEGVSSPAGRRMKVKDVSQYMIDAAKENPQLAQKLHDVLLESGVVAPRNLFSEVYSESMEATGEIKSVAESNDEKGKDFGTIQQGRNQSNLGPVRFLPPLPRPQSKAITHDLREHSGSGLGHLSEHCNIDGHSDSSHSETSTDYPRNVPVAVAAAAVVASSMVVAAAKSANSDSSTLELSAAAAAAVMATAAAVSRQFELDSLSNGDAGSGGLHGVDSGGERISDRSIGNESSKSDAAIDDVAECEILWEEITVAERIGLGSYGEVYRGDWHGTAVAVKKFIDQDITGEALEEFRSEVRMMRRLRHPNIVLFMGAVTRPPNLSIVTEFLPRGSLYRLIHRPNNQLDERKRLRMALDAARGMNYLHSCNPVIVHRDLKSPNLLVDKNWVVKVCDFGLSRMKVSTYLSSKSTAGTAEWMAPEVLRNEPADEKCDVYSYGVILWELFTLQQPWGKMNPMQVVGAVGFQHRRLDIPEFVDPGIADIIRKCWQTDPRLRPSFGEIMDSLKQLQKPIQRAAVPSSSALTTDEQEQ

>AtEDR1-5

MEMPGRRSNYTLLSQFSDDQVSVSVTGAPPPHYDSLSSENRSNHNSGNTGKAKAERGGFDWDPSGGGGGDHRLNNQPNRVGNNMYASSLGLQRQSSGSSFGESSLSGDYYMPTLSAAANEIESVGFPQDDGFRLGFGGGGGDLRIQMAADSAGGSSSGKSWAQQTEESYQLQLALALRLSSEATCADDPNFLDPVPDESALRTSPSSAETVSHRFWVNGCLSYYDKVPDGFYMMNGLDPYIWTLCIDLHESGRIPSIESLRAVDSGVDSSLEAIIVDRRSDPAFKELHNRVHDISCSCITTKEVVDQLAKLICNRMGGPVIMGEDELVPMWKECIDGLKEIFKVVVPIGSLSVGLCRHRALLFKVLADIIDLPCRIAKGCKYCNRDDAASCLVRFGLDREYLVDLVGKPGHLWEPDSLLNGPSSISISSPLRFPRPKPVEPAVDFRLLAKQYFSDSQSLNLVFDPASDDMGFSMFHRQYDNPGGENDALAENGGGSLPPSANMPPQNMMRASNQIEAAPMNAPPISQPVPNRANRELGLDGDDMDIPWCDLNIKEKIGAGSFGTVHRAEWHGSDVAVKILMEQDFHAERVNEFLREVAIMKRLRHPNIVLFMGAVTQPPNLSIVTEYLSRGSLYRLLHKSGAREQLDERRRLSMAYDVAKGMNYLHNRNPPIVHRDLKSPNLLVDKKYTVKVCDFGLSRLKASTFLSSKSAAGTPEWMAPEVLRDEPSNEKSDVYSFGVILWELATLQQPWGNLNPAQVVAAVGFKCKRLEIPRNLNPQVAAIIEGCWTNEPWKRPSFATIMDLLRPLIKSAVPPPNRSDL

>AtEDR1-6

MEMPGRRSNYTLLSQFSDDQVSVSVTGAPPPHYDSLSSENRSNHNSGNTGKAKAERGGFDWDPSGGGGGDHRLNNQPNRVGNNMYASSLGLQRQSSGSSFGESSLSGDYYMPTLSAAANEIESVGFPQDDGFRLGFGGGGGDLRIQMAADSAGGSSSGKSWAQQTEESYQLQLALALRLSSEATCADDPNFLDPVPDESALRTSPSSAETVSHRFWVNGCLSYYDKVPDGFYMMNGLDPYIWTLCIDLHESGRIPSIESLRAVDSGVDSSLEAIIVDRRSDPAFKELHNRVHDISCSCITTKEVVDQLAKLICNRMGGPVIMGEDELVPMWKECIDGLKEIFKVVVPIGSLSVGLCRHRALLFKVLADIIDLPCRIAKGCKYCNRDDAASCLVRFGLDREYLVDLVGKPGHLWEPDSLLNGPSSISISSPLRFPRPKPVEPAVDFRLLAKQYFSDSQSLNLVFDPASDDMGFSMFHRQYDNPGGENDALAENGGGSLPPSANMPPQNMMRASNQIEAAPMNAPPISQPVPNRANRELGLDGDDMDIPWCDLNIKEKIGAGSFGTVHRAEWHGSDVAVKILMEQDFHAERVNEFLREVAIMKRLRHPNIVLFMGAVTQPPNLSIVTEYLSRGSLYRLLHKSGAREQLDERRRLSMAYDVAKGMNYLHNRNPPIVHRDLKSPNLLVDKKYTVKVCDFGLSRLKASTFLSSKSAAGTPEWMAPEVLRDEPSNEKSDVYSFGVILWELATLQQPWGNLNPAQVVAAVGFKCKRLEIPRNLNPQVAAIIEGCWTNEPWKRPSFATIMDLLRPLIKSAVPPPNRSDL

>AtEDR1-7

MPHRTTYFFPRQFPDRGFDSFSLKNDHDKKKSSSNVGESFGFQRDNKSNGVGEDSNKEKESTVFSSNPLLSKSSAVSDLFSDDRKSEKKHQQQLAAFYEWLAEKKANLSRSSSTTTTHGRGVKPTRFSMSSDADEERELLLSSPADPAPLPATSSPDSIIDSARTVNIHERNIDRSFDREVSLPRMSSESSFAGSFFSGTTVDGNFSNFSSHTDARETSTTTLVSVNKEEEEVEVREQGKEQSLAQKSREGYYLQVTLAKWLSSQANLACESVHIQSTESISYRFWVSGCLSYSDKISDGFYSILGMDPYLWLMCNNSEDGKRIPSLLLLKETEPNDTSMEVVLIDRREDSRLKELEDKAHELYCSSDNMLVLVEKLGRLVAVYMGGNFQVEQGDLQKRWKLVSNRLKEFRKCIILPIGSLTMGLCRHRAILFKKLADYIGLPCRIARGCRYCKESHQSSCLVKIDDDRKLSREYVVDLIGEPGNVHDPDSSINGETQCQIPSPLQMSHLTDFSRPCVHSTSPCQTVESKTSRTLSENIQRSGSQGQVHKEFELPDNAGTVCCAHIDQTCCAKVSSMVLTESVLRALPLDIPNLSEEKIAPQETCKEETVLLEDPTAMKQPNLSVEPEIVEADTRKDKKGRLPVDAISPYLTIEPSLASDWLEVSWNELHIKERVGAGSFGTVHRAEWHGSDVAVKILSIQDFHDDQFREFLREVCKQAVAIMKRVRHPNVVLFMGAVTERPRLSIITEYLPRGSLFRLIHRPASGELLDQRRRLRMALDVAKGLNYLHCLNPPVVHWDLKSPNLLVDKNWTVKVCDFGLSRFKANTFIPSKSVAGTPEWMAPEFLRGEPTNEKSDVYSFGVVLWELITLQQPWNGLSPAQVVGAVAFQNRRLIIPPNTSPVLVSLMEACWADEPSQRPAFGSIVDTLKKLLKSPVQLIQMGGDKGVIPTKSAPIL

>AtEDR1-8

MEERRDDESSPTHQGSELAERVKLLSFESQGEALSKDSPRSVEQDCSPGQRASQHLWDTGILSEPIPNGFYSVVPDKRVKELYNRLPTPSELHALGEEGVRIEVILVDFQKDKKLAMLKQLITTLVSGSGTNPALVIKKIAGTVSDFYKRPTLESPSKLALEENAFLFENHGAQLLGQIKRGCCRARAILFKVLADTVGLESRLVVGLPSDGTVNCMDSNKHMSVIVVLNSVELLVDLIRFPGQLVPRSAKAIFMSHISPAGESDSAENDSCDSPLEPNSPLYERRDPESTEKDENLQFYRKLEGYPNASGSSLRSLMLRPSTAIERKLSNTSHSEPNVATVFWRRSRRKVIAEQRTASSSPEHPSMRRGRSMLSTGRNSFRDYTGEASSPSSSSTSEIRKTRRRSFRITPEIGDDIASAVREMYEKSKQNRLLQGREDENSSVIDNNVSGLHLDDELNSKKTMSLPSSPHAYRCQTFGRRGPSEFAVKDTWNKVVESSTLQNQPLLPYQEWDIDFSELTVGTRVGIGFFGEVFRGVWNGTDVAIKLFLEQDLTAENMEDFCNEISILSRVRHPNVVLFLGACTKPPRLSMITEYMELGSLYYLIHMSGQKKKLSWHRRLRMLRDICRGLMCIHRMKIVHRDLKSANCLVDKHWTVKICDFGLSRIMTDENMKDTSSAGTPEWMAPELIRNRPFTEKCDIFSLGVIMWELSTLRKPWEGVPPEKVVFAVAHEGSRLEIPDGPLSKLIADCWAEPEERPNCEEILRGLLDCEYTLC

>AtEDR1-9

MEERRDDESSPTHQGSELAERVKLLSFESQGEALSKDSPRSVEQDCSPGQRASQHLWDTGILSEPIPNGFYSVVPDKRVKELYNRLPTPSELHALGEEGVRIEVILVDFQKDKKLAMLKQLITTLVSGSGTNPALVIKKIAGTVSDFYKRPTLESPSKLALEENAFLFENHGAQLLGQIKRGCCRARAILFKVLADTVGLESRLVVGLPSDGTVNCMDSNKHMSVIVVLNSVELLVDLIRFPGQLVPRSAKAIFMSHISPAGESDSAENDSCDSPLEPNSPLYERRDPESTEKDENLQFYRKLEGYPNASGSSLRSLMLRPSTAIERKLSNTSHSEPNVATVFWRRSRRKVIAEQRTASSSPEHPSMRRGRSMLSTGRNSFRDYTGEASSPSSSSTSEIRKTRRRSFRITPEIGDDIASAVREMYEKSKQNRLLQGREDENSSVIDNNVSGLHLDDELNSKKTMSLPSSPHAYRCQTFGRRGPSEFAVKDTWNKVVESSTLQNQPLLPYQEWDIDFSELTVGTRVGIGFFGEVFRGVWNGTDVAIKLFLEQDLTAENMEDFCNEISILSRVRHPNVVLFLGACTKPPRLSMITEYMELGSLYYLIHMSGQKKKLSWHRRLRMLRDICRGLMCIHRMKIVHRDLKSANCLVDKHWTVKICDFGLSRIMTDENMKDTSSAGTPEWMAPELIRNRPFTEKCDIFSLGVIMWELSTLRKPWEGVPPEKVVFAVAHEGSRLEIPDGPLSKLIADCWAEPEERPNCEEILRGLLDCEYTLC

>AtEDR1-10

MGETGDDAGPSEQGPSNQTWWPSEFVEKFGSVYLGSQEETSSTKDSPRNLGQDGLPSSTASNILWSTGSLSEPIPNGFYSVIPDNRLKQLFNNIPTLEDLHALGDEGLKADVILVDFQKDKKLFRQKQLITKLVSGLNSKPATIIKKIAGLVADVYKQSTLQSPAKSTQSFENCGIQLLGQIKHGSCRPRAILFKVLADTVGLQSRLVVGLPSDGAAESVDSYSHISVTVLLNSVEMLVDLMRFPGQLIPLSTKAIFMSHISAAGESDSAENDSCDSPLEPNSPMFGYPEKFDHENAEKDENLSLHRKLDGSPNTSGPPSRNMLLRSASALERKLSFSQSESNMANEFWRQSRRKVIADQRTASSSPEHLSFRARTKSMLSGDKNLARDFTGDVATSSCKSVGGAKMETKRIRRRSISITPEIGDDIVRAVRAMNEALKQNRLSKEQGDDDSSPNSPNDRTESSHLQKNVSGFHLDAHDQVSGGRSTLSREPLDPQKAISLPSSPQNYRSQYEQSGSSHRNISHIWDKVLGSPMFQNKPLLPYEEWNIDFSELTVGTRVGIGFFGEVFRGIWNGTDVAIKVFLEQDLTAENMEDFCNEISILSRLRHPNVILFLGACTKPPRLSLITEYMEMGSLYYLLHLSGQKKRLSWRRKLKMLRDICRGLMCIHRMGIVHRDIKSANCLLSNKWTVKICDFGLSRIMTGTTMRDTVSAGTPEWMAPELIRNEPFSEKCDIFSLGVIMWELCTLTRPWEGVPPERVVYAIAYEGARLEIPEGPLGKLIADCWTEPEQRPSCNEILSRLLDCEYSLC

>AtEDR1-11

MGETGDDAGPSEQGPSNQTWWPSEFVEKFGSVYLGSQEETSSTKDSPRNLGQDGLPSSTASNILWSTGSLSEPIPNGFYSVIPDNRLKQLFNNIPTLEDLHALGDEGLKADVILVDFQKDKKLFRQKQLITKLVSGLNSKPATIIKKIAGLVADVYKQSTLQSPAKSTQSFENCGIQLLGQIKHGSCRPRAILFKVLADTVGLQSRLVVGLPSDGAAESVDSYSHISVTVLLNSVEMLVDLMRFPGQLIPLSTKAIFMSHISAAGESDSAENDSCDSPLEPNSPMFGYPEKFDHENAEKDENLSLHRKLDGSPNTSGPPSRNMLLRSASALERKLSFSQSESNMANEFWRQSRRKVIADQRTASSSPEHLSFRARTKSMLSGDKNLARDFTGDVATSSCKSVGGAKMETKRIRRRSISITPEIGDDIVRAVRAMNEALKQNRLSKEQGDDDSSPNSPNDRTESSHLQKNVSGFHLDAHDQVSGGRSTLSREPLDPQKAISLPSSPQNYRSQYEQSGSSHRNISHIWDKVLGSPMFQNKPLLPYEEWNIDFSELTVGTRVGIGFFGEVFRGIWNGTDVAIKVFLEQDLTAENMEDFCNEISILSRLRHPNVILFLGACTKPPRLSLITEYMEMGSLYYLLHLSGQKKRLSWRRKLKMLRDICRGLMCIHRMGIVHRDIKSANCLLSNKWTVKICDFGLSRIMTGTTMRDTVSAGTPEWMAPELIRNEPFSEKCDIFSLGVIMWELCTLTRPWEGVPPERVVYAIAYEGARLEIPEGPLGKLIADCWTEPEQRPSCNEILSRLLDCEYSLC

>AtEDR1-12

MGAKCCRGHRGSVVESNGSVILSLADLEVQATDLREMTVVEARDAAGPSEPRPPSPTLRPSEVEETSEPVCQGSLVETSNTDDAGPSEPNPPSPTLRPSEVEKIYVPVCQGSLAETSNLDQDGVSSYEASNIFWSTGSLSDPIPSGFYTVIPVERLMHFKSIPTLEEINALGEDRLKADAIFVDLKNDIQLVLIKEFVIKLVTGLDSDKVIKKIAGLVANIYKRKTLQSPARTLQYFDVQGFTLLGQIKHGSCRARAILFKVLADAVGLDSKLVMGFPTDLRFSASIDSYNHISAVVELNNVEMLVDLKRCPGQLKPFSPKAVYMAHISMAWQPDFVDNNPCASPLEPNSPMERSGPPSALQSGLSRSLGEPNIATEVLRRKVIKEPPPADFSGNSGAAESESKRTNGRCMNTPDLNNDIARATMMQSDLLKERGVDDSSPYSPDEKNVSGFQLDSHDLVSGECSTVYPRKSISLPSSPRSYQIQLSERSEHSPQEISHIWNEVLESPMFQNKPLLPFEEWNIDFSKLKVGASVGSGTSGVVCRGVWNKTEVAIKIFLGQQLTAENMKVFCNEISILSRLQHPNVILLLGACTKPPQLSLVTEYMSTGSLYDVIRTRKKELSWQRKLKILAEICRGLMYIHKMGIVHRDLTSANCLLNKSIVKICDFGLSRRMTGTAVKDTEAAGTPEWMAPELIRNEPVTEKSDIFSFGVIMWELSTLSKPWKGVPKEKVIHIVANEGARLKIPEGPLQKLIADCWSEPEQRPSCKEILHRLKTCEIPIC
