# Supplementary material for: Identification and expression analysis of EDR1-like genes in tobacco (Nicotiana tabacum) in response to Golovinomyces orontii
Source: PeerJ. 2018 Jul 10;6:e5244. doi: 10.7717/peerj.5244 (PMC6044316; doi:10.7717/peerj.5244)
Supplement: Supplemental Information 5 [file peerj-06-5244-s005.docx]

>NtEDR1-1A

MKHIFKKLHHPNRSNDAQSTSSAVSTSSPPAVSSSSSASCASDNRNSSSVVQSPSTPSTTSSASTAAAAPVSAGGGGGNISTIIRQQDYYTSEEEYQVQLALALSVSSSQAQDSFPCDVNSSNGQTLGGGRTAADLARDREDAAADLLSRQYWDYGVLDYEEKVVDGFYDVYTLFTDPASRGKMPSLTELETIPGSSDFEGVIINRRIDPSLEELMQIAHCITLDCPASEINLLVLRLSELVTEHLGGPVKDANIILAKWMEISNELRTSLHTSVLPIGSLKVGLSRHRALLFKVLADHVGIPCRLVKGSHYTGVEDDAVNIVNLPNDSEFLVDLMGAPGTLIPADVLSTKDASFKSYGPKLNKIPSFPSNNDSRVSYPRLNLLSGQNSGLGDDFSNRSRPEKTDSVHSISDAGGSSTPGSSAISKRPSSSNQVDWTSPLAIGTSLYKGGRGPNAAGDGLRLNVNVVPYDQNNPEDPKNLFADLNPFQVKGSGNTLIQKNPARNKVSELQQPKNTLVTGRPPAPMMWKNRYAHNEVPWKNDSDSEALFPKKSCGSSGYNTSSIASTSSNIPQKSSPDTTSRFHGNSHPAYRGNEGVTSTQDNSSKLSADLEFRRLSVQDGQSNNRETSQWEGHILQTDELNRTQAHGQAIILESDHIRNLQAQPTGTNIKLKEPEYPTSSGDLGRSQVDPVFDDVGDCEIPWEDLVIGERIGLGSYGEVYHADWNGTEVAVKKFLDQDFSGAALAEFKREVRIMRRLRHPNVVRFMGAITRPPHLSIITEFLPRGSLYRIIHRPHCQIDERRRIKMALDVAKGMDCLHTSNPTIVHRDLKSPNLLVDKNWTVKVCDFGLSRLKHNTFLSSKSTAGTPEWMAPEVLRNEPSNEKCDIYSFGVILWELATLRLPWSGMNPMQVVGAVGFQNKRLEIPKELDPIVARIIWQCWQTDPNLRPSFAQLTVALAPLQRLIIPSYVDQPSSHLPQEISVNSTP

>NtEDR1-1B

MKHIFKKLHHPNRSNDAQPTSSAVSSSSPAAVSSSSSASCATDHRNSSSVAQSPSTPSTTSSASTAATAPASAGGGGGNISTINRQQDYYTSEEEYQVQLALALSVSSSQAQDSFPCDVNSSNGQILGGGRTAADLARDREDAAADLLSRQYWDYGVLDYEEKVVDGFYDVYTLFTDPASRGKMPSLTELETIPGSSDFEGVIINRRIDPSLEELMQIAHCITLDCPASEINLLVLRLSELVTEHLGGPVKDANIILAKWMEISNELRTSLHTSVLPIGSLKVGLSRHRALLFKVLADHVGIPCRLVKGSHYTGVEDDAVNIVKLPNDRKYCACLTMVDSVYNSFCCRSCCPLNQFFAFSEFLVDLMGAPGTLIPADVLSAKDASFKSYGPKLNKIPSFSSNNDSGVSYPRLNLLSGQISGLGDDFSNRSKPEKIESVHSISDAGGSSTPGSSAISKRPSSSNQVDWTSPLAIGTSLYKGGRGPNAAGDGLRLNVNVVPYDQNNPEDPKNLFADLNPFQVKGSGNTVMQKNPARNKVSELQQPKNTLVTGRPPAPMMWKNRYAHNEVPWKNDSDSEALFPKKNCGSSGYNTSSIASTSSNIPQKSSPDTSRLQGNSHPAYRGNEGVTSTQDTSSKLVAEHEFRRLSVQDSQNNNRETSQWEGHILQTDELNRTQAHGQAIILESDHIRNLQAQSIGTNIKLKEPEYPTSSGDAGRNQVDPVFDDVGDCEIPWEDLVIGERIGLGSYGEVYHADWNGTEVAVKKFLDQDFSGAALAEFKREVRIMRRLRHPNVVRFMGAITRPPHLSIITEFLPRGSLYRIIHRPHCQIDERRRIKMALDVAKGMDCLHTSNPTIVHRDLKSPNLLVDKNWTVKVCDFGLSRLKHNTFLSSKSTAGTPEWMAPEVLRNEPSNENMLVSVRLVVTACFVLAEFYAIIGLIFWSLDRCDIYSFGVILWELATLRLPWSGMNPMQVVGAVGFQNKRLEIPKELDPVVARIIWQCWQTDPNLRPSFAQLTVALAPLQRLIIPSYVDQPSSHLPQEISVNSTP

>NtEDR1-2

MKNFLKKLHIGSNQSEDSEGSTSSSKSKRLSDVSSPDGNSNSRNSQGSDNKTFSAISGWLNSVTNRKSPSPPSSSNVSRGNRMEHSDSVTIGELDAALDAVQRDSESSNSRDPGVEEEYQIQLALELSAKEDPEAVQIEAVKQISLGSSAPENAPAEVVAYRYWNYNALSYDDKILDGFYDLYGVLMESNSSKMPSLIDLQRTEVSDHISWEAILVSKAADSKLLKLEQRALEIAVGLRSKLIDFSDSSLMQKLAVLVSDHMGGPVADPDSMLLAWRSLSFNLKATLGSMVLPLGSLTIGLARHRALLFKVLADSVGIPCRLVKGKQYTGSDDVAMNYVKIDGREYIVDLMADPGTLIPSDTCGIHGDYEESILSISPSSRDVDSHPGSSSSGVASSLEDHSDYGMADKRSRFAECTTAGNEPPSSSNLEQQIKAEKGCYNTFHDFTKEQGQETSSRAGHARSPFTHARSPSWTEGVSSPAVRRMKVKDASQYMIDAAKENPQLAQKLHTVLLESGVIAPPNLFAEMYPEQLDVSHVEGKSRLEEIESKERGEFQFRGQSDANRARFLPPLPYHGSYSKGNARGSFEPQPDVREVGEQQVSRQSEVAPLKPMKQMPVAAAAAAAAAVVASSMVVAAAKTNSHTDLPVAAAATATAAAVVATTAAVSRQYEALGDCGRVDGDADTAIYEHQRSGDQEHEALGANSEGERISDRSSGNDSAKSDVTLDDVADCEIPWDDIALGDRIGLGSYGEVYRGEWHGTEVAVKKFLDQDITGDSLEEFRSEVRIMKRLRHPNVVLFMGAVTRSPHLSIVTEFLHRGSLYRLIHRPNNQLDERRRLRMALDAARGMNYLHNCTPMIVHRDLKSPNLLVDKNWVVKVCDFGLSRMKHSTFLSSRSTAGTAEWMAPEVLRNEPSNEKCDVYSFGVILWELCTLQQPWGGMNPMQVVGAVGFQHRRLDIPEDMDPAVADIIRKCWQTDPRLRPSFAEIMAALKPLQKPISSQAPRPPAGRGPVKGQPSRIVEDPPADQS

>NtEDR1-3

MKNFLKKLHIGSNQSEDSEGSTSSSKSKRLSDVSSPERNSNSRNSQGSDNKPFSAISGWLNSVTNRKSPSPPSSSNVSRGNRMEHSDSVTIGELDAALDAVQRDSESSNSRDPSVEEEYQIQLALELSAKEDPEAVQIEAVKQISLGSSAPENAPAEVVAYRYWNYNALSYDDKILDGFYDLYGVLMESNSSKMPSLIDLQRTKVSDHISWEAILVSKAADSKLLKLEQRALEIAVGLRSKLIDFSDSSLMQKLAVLVSDHMGGPVADPDSMLLAWRSLSFNLKATLGSMVLPLGSLTIGLARHRALLFKVLADSVGIPCRLVKGKQYTGSDDVAMNYVKIDGREYIVDLMADPGTLIPSDTSGIHGDYEESILSISPSSKDVDSHPGSSSNGIASSLEDHSDYGTADKRSRFAESTTAGNESPSSSNSEQQIKAEKGCYKTFNDFTKEQGQETSSRAGHARSPFTHARSPSWTEGVSSPAVRRMKVKDASQYMIDAAKENPQLAQKLHTVLLESGVIAPPNLFAEMYPEQLDVSHVEGKSRLEEIERKERGEFQFRGQSDANRARFLPPLPYHGSYSKGNACGSFEPQPDVREVGEQQVSRQSEVAPSKPMKQMPVAAAAAAAAAVVASSMVVAAAKTNSHTDLPVAAAATATAAAVVATTAAVSRQYEALGDYGRVDGDADTAIYEHQRSGDQEHEALGANSEGERISDRSSGNDSAKSDVTLDDVADCEIPWEDIALGERIGLGSYGEVYRGEWHGTEVAVKKFLDQDITGDSLEEFRSEVRIMKRLRHPNVVLFMGAVTRSPHLSIVTEFLHRGSLYRLIHRPNNQLDERRRLRMALDAARGMNYLHNCTPMIVHRDLKSPNLLVDKNWVVKVCDFGLSRMKHSTFLSSRSTAGTAEWMAPEVLRNEPSNEKCDVYSFGVILWELCTLQQPWGGMNPMQVVGAVGFQHRRLDIPEDMDPAIADIIRKCWQTDPRLRPSFAEIMAALKPLQKPISSQAPRPRAGRGPIKGQPSRIVEDPTADQD

>NtEDR1-4

MKNLLKKLHLVPNESVDSEGSTSSAKTKRLSDVSFPDNKPFSAISGWLNSVTNRHNSPSPPSSSNVSRGNNNRMEPCDSASSSGLDAALDSVRRDSESSNSREPDIEEEYQIQLALELSAKEDPEAVQIEAVKQISLGSCAPENTPAEIVAYRYWNYNALSFDDEILDGFYDLYGIMTESNSSKMPSLVDLQRTPVSDQIAWEAILVNRAADSKLLMLEQKALEISVKVRSESIGFTGGNLVQTLAMLVSEHMGGRVGDPESMLVAWRSLSYNLKATFGSMVLPLGSLTIGLARHRALLFKVLADCVGLPCRLVKGQEYTGSDEVAMNYVKIEDGREYIVDLMADPGTLIPSDTSGTQVDYEESILSISPSYKDVDSHMGSSSSGVACSSEDHSEYGTAERRSRFRESSTENASPSSSNNSEKQIKAEKGCNNSSKEFTKLRTVKKGQRQETSPGTGHARSPYTHARSPSWTEGVSSPAVRRMKVKDVSQYMIDAAKENPQLAQKLHDVLLESGVVAPPNLFTEVYTEELDVSPVEGKSGPEDMESKGSDEIEKNKSQADLDRNSFLPPLPYHGMSKANPLRPFDPHLDGGEVSGHQVSPHSELAAVKFTKSMPVAAAAAAAAAVVASSMVVAAAKTTYGSKADLPVAAAVTATAAAVVATTAAVTKQYENLEPSAHSPDSPAFFLNLIDSGRVDGDADGAVYEQRGSGDQVHEALGVNSESERISDRSTSNDSVKSDVTLDDVADCEIPFEDITLGERIGLGSYGEVYRGDWHGTEVAVKKFIDQDITGESLEEFKSEVRIMKRLRHPNVVLFMGAVTRPPNLSIITEFLHRGSLYRLIHRSNNQLDERRRLRMALDAARGMNYLHNCTPMVVHRDLKSPNLLVDKNWVVKVCDFGLSRMKQSTFLSSRSTAGTAEWMAPEVLRNEPSDEKCDVYSYGVVLWELCTLQQPWGGMNPMQVVGAVGFQHRRLDIPDDMDPAIADIIRKCWQTDPKLRPSFAEIMAALKPLQKPITSSQVMKPLGNRGQEKDRS

>NtEDR1-5

MSKMKHLLRKLHIGGGVADQHPHHPPHHTPPPPPHQSPPPLLDPNQQTTNRFEQSGSTSSVSPTQSGSTSSALARAAAESNSGSVSDSADFNYFEEEFQVQLALAISVSDPNSREDPETAQIKAAQEISLGCSPLENPVEFLSLRYWNYNVVNYDEKVVDGFYDVYGINSSGVVQGKMPLLVDLKAVSVLDNVNYEVILVNRAADMELRQLEERVMGGPVNDAEEMSKRWTARSYELRISLKSIILPLGCLDIGHSRHRALLFKVLADRINLPCKLVKGSYYTGTDDGAVNLIKFDNGSEYIIDLMGAPGALIPTEASSGQLQSYAVDVHSVTPLPAGGTIISIPVFDTQTGTKSGSVTAAHGTANTWISRAEPAFYCIEAKGGSGNSSVRPGSTQFEHDCGNLLPSSTRLCDTSAVSHDNTSMAQITQAREAYEHVNGPAENTEVKLQDVLPESQMYLPSDLILGVVAGKNQLSENRVVDTRQSSENNKQSLIAFTGMQFPYSITYETGILQHKQEYTVTAPGDNALNDTSGESFPPVRCRMDETIQTGDGAFLGFYFIHLRDKCYRENFGNISDNNCTYKDKESASKAREIVTCIQSKSYTVQKEQLPMLRGVAEWEIPWEDLHVGERIGIGSFGEVYRAEWNGTEVAVKKFMNQDITSDALAQFKCEIEIMLRLRHPNVVLFMGAVTRPPNLSILTEFLPRGSLYRLLHRPNIQIDEKRRMRMALDVAKGMNYLHTSNPVIVHRDLKTPNLLVDKNWVVKVCDFGMSRMKHHTFLSSKSTAGTAEWMAPEVLRNEPSNEKSDVYSFGVIFWELTTLKVPWSGMNSMQVVGAVGFQGRRLDIPATVDPIVAEIISDCWNQNSQARPSFGQIITRLKCLQRLNVQGFETCTNQQ

>NtEDR1-6

MQNYNVVNYDEKVVDGFYDVYGINSSGVVQGKMPLLVDLKAVSVLDNVNYEVILVNRAADMELRQLEERVYCRALKKVPVTSFLVEKIAELVANRMGGPVNDAEEMSKRWTARSYELRISLNSIILPLGCLDIGHSRHRALLFKVLADRINLPCKLVKGSYYTGTDDGAVNLIKFDNGSEYIIDLMGAPGALIPTEASSGQLQSYAVDVHSVTPLPAGGTIISIPVFDTQTGTESGSVTAAHGTANTWISRAEPAFYCIEAKGGSGNSSVRTGSTKFEHDCGNLLPSSARLCDTSAVSHDNTSMAQITQAREAYEHVNCPAENTDVKLRDVFPESQMYLQSDLILGVVAGKNQLSENRVVGTRQSSENNNQSLIAFTGMQFPYSITYETGILQPKQEYTVTAPGDNALNDTSGDKCYREKFGNISDNNCAYKDKESASKAREIVTCIQSKSYTVQKEQLPMLRGVAEWEIPWEDLHVGERIGIGDHGHRDAYSRRSQAKLLEPPTEEVAVKKFMNQDITSDALAQFKCEIEIMLRLRHPNVVLFMGAVTRPPNLSILTEFLPRGSLYRLLHRPNIQIDEKRRMRMALDVAKGMNYLHTSNPVIVHRDLKTPNLLVDKNWVVKVCDFGMSRMKHHTFLSSKSTAGTAEWMAPEVLRNEPSNEKSDVYSFGVIFWELTTLKVPWSGMNSMQVVGAVGFQGRRLDIPATVDPIVAEIISDCWNQNSQARPSFGQIITRLKCLQRLNVQGFETCTNQQ

>NtEDR1-7

MPGRRSNYTLLSQVPDDNFLPPPSKYGGGGGGAQYYESHSGEKSNKGKGVGDNRGYDWDLIDHRMMQAPTTSRIGAAAPFPVSIGLQRQSSGSSFGESSISGEYYMPSSLSNAEASFGYPNDGGGGGAELRMKPLDAANLCGSSSKSWAQQTEESYQLQLALALRLSSEATCADDPNFLDPVPDESASRASASAASVETVSHRFWVGLETMYVRIQGEMKYNLEKVNGCLSYYDRVPDGFYLIHGMDPYVWIVCSDLQESARIPSIESLRAVDPSVVPSLEVILIDRRSDPSLKELQNRIHNLSPSCITTKEVVDQLAKVVCNHMGGTASAGEDELVPMWKECSDDLKDCLGSVVLPIGSLSIGLCRHRALLFKVLADIIDLPCRIAKGCKYCNRADASSCLVRFGLDREYLVDLIGNPGCLCEPDSSLNGPSSISISSPLRFPRFREVEPTIDFRSFAKQYFSDCQSLNLVFEDPSAGAAVDGDSGQTDRNNMERNSVVPSSSNHDEISRLPVPPVGHGDTQPLMALSDPRGRGNDMRFLDGGSQLVPAKQSRDLALEVEEFDIPWEDLVLKERIGAGSFGTVHRADWNGSDVAVKILMEQDFHAERFKEFLREVAIMKRLRHPNIVLFMGAVTQRPNLSIVTEYLSRGSLYRLLHKPGAREVLDERRRLSMAYDVAKGMNYLHKRNPPIVHRDLKSPNLLVDKKYTVKVCDFGLSRLKANTFLSSKSAAGTPEWMAPEVLRDEPSNEKSDVYSFGVILWELATLQQPWSNLNPAQVVAAVGFKGKRLEIPRDLNPQVASIIEACWAKEPWKRPSFSVIMDMLRPLIKPPVTPPQPGRTDMQLLT

>NtEDR1-8

MPGRRSNYTLLSQVPDDNFLPPPSKYGGGGGGAQYYESHSGEKSNKGKGVGDNRGYDWDLIDHRMMQAPTTSRIGAAAPFPVSIGLQRQSSGSSFGESSISGEYYMPSSLSNAEASFGYPNDGGGGGAELRMKPLDAANLCGSSSKSWAQQTEESYQLQLALALRLSSEATCADDPNFLDPVPDESASRASASAASVETVSHRFWVGLETMYVRIQGEMKYNLEKVNGCLSYYDRVPDGFYLIHGMDPYVWIVCSDLQESARIPSIESLRAVDPSVVPSLEVILIDRRSDPSLKELQNRIHNLSPSCITTKEVVDQLAKVVCNHMGGTASAGEDELVPMWKECSDDLKDCLGSVVLPIGSLSIGLCRHRALLFKVLADIIDLPCRIAKGCKYCNRADASSCLVRFGLDREYLVDLIGNPGCLCEPDSSLNGPSSISISSPLRFPRFREVEPTIDFRSFAKQYFSDCQSLNLVFEDPSAGAAVDGDSGQTDRNNMERNSVVPSSSNHDEISRLPVPPVGHGDTQPLMALSDPRGRGNDMRFLDGGSQLVPAKQSRDLALEVEEFDIPWEDLVLKERIGAGSFGTVHRADWNGSDVAVKILMEQDFHAERFKEFLREVAIMKRLRHPNIVLFMGAVTQRPNLSIVTEYLSRGSLYRLLHKPGAREVLDERRRLSMAYDVAKGMNYLHKRNPPIVHRDLKSPNLLVDKKYTVKVCDFGLSRLKANTFLSSKSAAGTPEWMAPEVLRDEPSNEKSDVYSFGVILWELATLQQPWSNLNPAQVVAAVGFKGKRLEIPRDLNPQVASIIEACWAKEPWKRPSFSVIMDMLRPLIKPPVTPPQPGRTDMQLLT

>NtEDR1-9

MEMPGRRSNYTLLSQVPDDNFLPPPSKYGGGGGGAQYYESHSGEKSNKGKGAGDNRGYDWDLIDHRMMQAPTTNRIGAAAPFPGSIGLQRQSSGSSFGESSISGEYYMPSSLSNAEASFGYLNDGGGGGGGAELRMKPLDAANLCGSSSKSWAQQTEESYQLQLALALRLSSEATCADDPNFLDPVPDESASRASASAASVETVSHRFWVNGCLSYFDKVPDGFYLIHGMDPYVWIVCSNLQESARIPSIESLRAVDPSVVPSLEVILIDRRSDPSLKELQNRIHNLSASCITTKEVVDQLAKVVCNHMGGTASAGEDELVPMWKECSDDLKDCLGSVVLPIGSLSIGLCRHRALLFKVLADIIDLPCRIAKGCKYCNRADASSCLVRFGLDREYLVDLIGNPGCLCEPDSSLNGPSSISISSPLRFPRFREVEPTTDFRSLAKQYFSDCQSLNLVFEDPSAGAAVDGESGQTDRNNMERNSVVPSSSNRDEISRLPVPPINAWKLDRDMVPVKHVPPVGHGDTQPLMALSDPRERGNDMRFLEGGSQLVPAKQSRDLALEVEEFDIPWEDLVLKERIGAGSFGTVHRADWNGSDVAVKILMEQDFHAERFKEFLREVAIMKRLRHPNIVLFMGAVTQRPNLSIVTEYLSRGSLYRLLHKPGAREVLDERRRLSMAYDVAKGMNYLHKRNPPIVHRDLKSPNLLVDKKYTVKVCDFGLSRLIENTFLSSKSAAGTPEWMAPEVLRDEPSNEKSDVYSFGVILWELATLQQPWSNLNPAQVVAAVGFKGKRLEVPRDLNPQVASIIEACWAKEPWKRPSFSAIMDMLRPLIKPPVTPPQPGRTDMQLLT

>NtEDR1-10

MEVSGRRSSYTLLNQIPDDNFVLPPPPKFSAGADLIEDRMMQSQSRVGSLQLPGSGGSHRQSSEGSFGGSSISGEYYAETSFGLRNDGCGSSAAARSWALQTEESYQLQLALAIRLSSEATCADNPNFLGPAADESASRDSDSSASAETMSHRLWINGCLSYFDKVPDGFYWIYGMDPYVWTVCSVLQESGRIPSIESLKAVDPTVAPSVEVILIDRCNDPSLKELQIGILSMSASCISVEEVVDQLAKLVCDHMGGAAPAGEDDLVSMSKERSDDLKDCLGTIVLPIGSLSVGLCRHRALLFKVLADIIDLPCRIAKGCKYCNRSDASSCLVRLGLDREYLVDLVGKPGVLCEPDSLINGPSSISIPSPLRFPRYRQVEPTIDFRSLAKQYFLDSQSLNLLFDDSSAGSAADGVAGQSDRSCMDRNNAVPSSSNRDAISRLPLPPSNAWKKGRDKESQLSKMYNPSSMLNSMNVDKDLVPVKHVPPIGEDAQPLMALSHPRADTINDGELLSDVEEFNIPWNDLVLKERIGAGSFGTVHRADWDGSDVAVKILMEQNFHAEGFNEFLREVAIMKRLRHPNIVLFMGAVIQPPNLSIVTEYLSRGSLYRLLHRPGARELLDERRRLCMAYDVANGMNYLHKRNPPIVHRDLKSPNLLVDKKYTVKVCDFGLSRFKANTFLSSKTAAGTPEWMAPEVLRDEPSNEKSDVYSFGVILWELATLQQPWSNLNAPQVVAAVGFRGKRLDIPSDLNPQVATIIEACWAKCVACTRPQVHAF

>NtEDR1-11

MEEIPDEVELPEHRYPNTAWWPSDYVEKLGSVSLDGKEEIMRNREPTEREYDRLSSQTASQILWKTGTLSEPIPNGFYSVVPEKRLKELFEDIPTLDELHALEPEGLGADVIVVDTKKDKKLSMLKQLIVALVKGLNSTPAAMIKKIAGLVSDVYKRPNSELCHTKASHDEASHISDNRGVQMLGQIKHGSCRPRAILFKLLADTVGLESRLVVGLPAEGASECEESYKHMSILVVLNSVELLVDLMRFPGQLIPRTTKAIFLTHIAAGESDSAENDSCDSPLEPNSPLYGVSERNDTDSSEKDDILQYQRRFEASSGAAGYSLRNMMLRSNTSIDRKLSLSHSEPNTASTVWRRGRRKVITEPRTASSSPEHPSFRAHGRSMLSGDNKTFRDYSDDVATSRSEGASTSETRRLRRRSISITPEIGDDIVRAVRAMNEALKQNRLLKEQEENTSFHASNNRDCASDLKKDISSQKAMSLPSSPHELRRQAPESSGPDRTNNELFSTWNRILESHMYQNKPLLPFEEWNIDFSELTVGTRVGIGFFGEVFRGKWNGTEVAIKVFLEQDLTAENMEDFCNEISILSRLRHPNVILFLGACANPPRLSMVTEYMEMGSLYYLTHLSGQKKRLSWQKRLNMLRDICRGLMCLHRMKIVHRDLKSANCLVNKRWTVKICDFGLSRIMTDASMKDLASAGTPEWMAPELIRNEPFTEKCDIFSFGVIMWELCTLNRPWEGIPPDRVVYAVANEGARLEIPEGPLGRLIADCWAEPNERPSCEEILTRLLDCEYSLC

>NtEDR1-12

MPHRTTYFFPRQFPDRGFDASAKFVNDDHEKKISTVVEDQIKSGKSSDNVTSKQLTSDHGKETNNNASFSYGHRDKIHGKQLSAFVNWLAEKNKKGKSIQNHVKIKLDDVDGDDEHELLLPAPPEAVPVHELVACHVAEAEAEQKQRQGSTFDRKVSLQRLSSSGSNFSCAGKGNFERQTSLQRLSSWGSTSYAGSLFSGTTLDGNWPSTGVKDTQTDQSTTREVEEVVAAEEEVERVDSEGSLMQKSKESYYLQLTLAKKLVEQAMLASGEPMLLQECRSTKGLGGSSDAQTVSYRLWVSGSLSYADKISDGFYNILGMNPYLWVMCNETEDGRRTPSLMALKEIEPGDTSMEVVLIDRRGDSKLRELEDKAQEIYFAAENTLVLAEKLGKLVAVGSFPVEQGDLHQRWKVVSKRLKDLQKCIVLPIGNLSSGLCRHRAILFKKLADYVGLPCRIARGCKYCVADHRSSCLVKIEDDRRLSREFVVDLVGDPGNVHGPDSSINGGVLAPVPSPLQVSHLKEYQQPYVDSDISNQLLLSNDTFGPAENALHTDPHIEGDHVSEFAVSDKPKLPNDALCRSYQALEVEPSEVLVAAETAGDEYSRPREDKIIIRQTYKEEVVLSKKSPICPGRPPKATLLANIDAMEARGRTGKREKPAATNPRYLNLEPSLAMDWLEISWDELQMKERVGAGSFGTVHRAEWHGSDVAVKLLTVQDFHDDQLKEFLREVAIMKRVRHPNVVLFMGAVTKRPHLSIVTEYLPRGSLYRLIHRPAAGELLDQRRRIRMALDVAKGINYLHLLNPPVVHWDLKSPNLLVDKNWNVKVCDFGLSRFKANTFISSKSVAGTPEWMAPEFLRGEPSNEKSDVYSFGVILWELVTMQQPWNGLGPAQV

>NtEDR1-13

MLIKYAEFAGRGISLRHMLHIFASHFLQGSMIHMRHRGTKSLLVKIDNKVNRRFWESYFYLRTKHLVADKARFPKTWNFAPERLPPPLVNDIREWVGAILPYTLGIHEWVSFHEKYGRKPLTRRVRRTRSPSLAFCQPVPPTQPVPRAAIRPAPRATTQSASAAGAVCTEATPRSKIPVPTVHSTSKFSRIDSGEAPSKRQRVALEVAPQAETSSRGDISLAVPEIDGGANPAVEATPVVSGQQADVVERRSPETAVAVLGVPSTTWPNRTSSSAKERGKCVMVDYYESESDVNPNDVRMFEEGFTGTMVRAGEIGALQDDSDTELSHGVAAMGLRSYMLEIESAHRAETRAKIFLKMLEKYRRYHNKYREMHERLRAGTGTQSIGGVRIHADEAVDVGKPPPLCQLACLSLQCQGNGIAIKLSTDMEETRDEVGPSEQRSPGAAWWPSDFVEKFGSVTLDSKEENLRNKEPRENEVYDSLPCQTASQILWKTGTLSEPIPNGFYSVVAEKTLKELFEDIPTFDELHSLELEGLRADIILVDTEKDKKLSMLKQLIVALVKGLSSNPAAIIKKIAGLVSDFYKRPNSELSPAKAASEESSHISDNQGIQMLGQIKHGSCRSRAILFKVLADTIGLESRLVVGLPTEGASECVDSDKHMSVIVVLNSVELLVDLIRFPGQLIPRSTKAIFMTHISAAGESDSAENDSCDSPLEPNSPLYGFSERIDPESSEKDDTLQCQRRLEASSNAAGPSLRSMMLRSNTSIDRKLSLSHSEPNIATAAWRRSRRKVITEQRTASSSPEHPSFRARARSMLSGDNKTFRDYSDDVATSRSTGASTSEPRRLRRRSISITPEIGDDIVRAVRAMNEALKQNRREQGENSSLPHTSNDRGGALDHQKNVSDFHHDDHEILGAQSSLFALSREHMNSQKAISLPSSPNEFRRQAPERRGQVNDETVSTWNRILESPMFLNKPLLPFEEWNIDFSELTVGTRVGIGFFGEVFRGIWNGTDVAIKVFLEQDLTAENMEDFCNEISILSRLRHPNVILFLGACTKPPCLSMVTEYMEMGSLYYLIHLSGQKKRLSWRRRLKMLRDICRGLMCIHRMKIVHRDLKSANCLVNKHWTVKICDFGLSRIMTDAPMRDSTSAGTPEWMAPELIRNESYTEKCDIFSLGVIMWELCTLKRPWEGVPPERVVYAVANEGSRPEIPEGPLGQLIADCWAEPNKRPSCEEILTRLLECEYSLC

>NtEDR1-14

MEETRDEVGPSEQRSPGAAWWPSDFVEKFGSVTLDSKEENLRNKEPRENEVYDSLPCQTASQILWKTGTLSEPIPNGFYSVAAEKRLKELFEDIPTFDELHSLELEGLRADIILVDTEKDKKLSMLKQLIVALVKGLSSNPAAIIKKIAGLVSDFYKRPNSELSPAKAASDESSHISDNRGIQMLGQIKHGSCRSRAILFKVLADTIGLESRLVVGLPTEGASDCVDSDKHMSVIVVLNSVELLVDLMRFPGQLIPRSTKAIFMTHISAAGESDSAENDSCDSPLEPNSPLYGFSERIDPESSEKDDTLQCQRRLEASSNAAGPSLRSMMLRSNTSIDRKLSLSHSEPNIATAAWRRSRRKVITEQRTASSSPEHPSFRARARSMLSGDNKTFRDYSDDVATSRSTGASTSEPRRLRRRSISITPEIGDDIVRAVRSMNEALKQNRREQGENSSLLHTSNDRGGALDHQKNVSDFHHDDHEILGAQSSLFALSREHMNSQKAISLPSSPNEFRRQAPERRGHVNDETVSTWNRILESPMFLNKPLLPFEEWNIDFSELTVGTRVGIGFFGEVFRGIWNGTDVAIKVFLEQDLTAENMEDFCNEISILSRLRHPNVILFLGACTKPPRLSMVTEYMEMGSLYYLIHLSGQKKRLSWRRRLKMLRDICRGLMCIHRMKIVHRDLKSANCLVNKHWTVKICDFGLSRIMTDAPMRDSTSAGTPEWMAPELIRNESYTEKCDIFSLGVIMWELCTLKRPWEGVPPERVVYAVANEGSRPEIPEGPLGQLIADCWAEPNKRPSCEEILTRLLECEYSLC

>NtEDR1-15

MEVSGRRSSYTLLNQIPDDNFVLPPPPKFSAGADLIEDRMMQSQSRVGSLQLPGSGGSHRQSSEGSFGGSSISGEYYAETSFGLRNDGCGSSAAARSWALQTEESYQLQLALAIRLSSEATCADNPNFLGPAADESASRDSDSSASAETMSHRLWINGCLSYFDKVPDGFYWIYGMDPYVWTVCSVLQESGRIPSIESLKAVDPTVAPSVEVILIDRCNDPSLKELQIGILSMSASCISVEEVVDQLAKLVCDHMGGAAPAGEDDLVSMSKERSDDLKDCLGTIVLPIGSLSVGLCRHRALLFKVLADIIDLPCRIAKGCKYCNRSDASSCLVRLGLDREYLVDLVGKPGVLCEPDSLINGPSSISIPSPLRFPRYRQVEPTIDFRSLAKQYFLDSQSLNLLFDDSSAGSAADGVAGQSDRSCMDRNNAVPSSSNRDAISRLPLPPSNAWKKGRDKESQLSKMYNPSSMLNSMNVDKDLVPVKHVPPIGEDAQPLMALSHPRADTINDGELLSDVEEFNIPWNDLVLKERIGAGSFGTVHRADWDGSDVAVKILMEQNFHAEGFNEFLREVAIMKRLRHPNIVLFMGAVIQPPNLSIVTEYLSRGSLYRLLHRPGARELLDERRRMCMAYDVVCDFGLSRFKANTFLSSKTAAGTPEWMAPEVLRDEPSNEKSDVYSFGVILWELATLQQPWSNLNAPQVVAAVGFRGKRLDIPSDLNPQVATIIEACWAKCVACTRPQVHAF

>NtEDR1-16

MPHRTTYFFPRQFPDRGFDASAKFVNDDHEKKISTVVEDQIKSGKSSDNVTSKQLTSDHGKETNNNASFSYGHRDKIHGKQLSAFVNWLAEKNKKGKSIQNHVKIKLDDVDGEDEHELLLPAPPEAVPVHELVDCHVAEAEQKQRQGSTFDRKVSLQRLSSSGSNFSCVGKGNFERQTSLQRLSSWGSTSYAGSLFSGTTLDGNWPSTGVKDTQTDQSTTREVEEVVAAEEEVERVDSEDSLMQKSKESYYLQLTLAKKLVEQAMLASGEPMLLQECRSTKRLGGSSDAQTVSYRLWVSGSLSHADKISDGFYNILGMNPYLWVMCNETEDGRRIPSLMALKEIEPSDTSMEVVLIDRRGDSRLRELEDKAQEIYFAAENTLVLAEKLGKLVAVGSFPVEQGDLHQRWKVVSKRLKDLQKCIVLPIGNLSSGLCRHRAILFKKLADYVGLPCRIARGCKYCVADHRSSCLVKIEDDRRLSREFVVDLVGDPGNVHGPDSSINGGVLAPVPSPLQVSHLKEYQQPYVDSDISNQLLLSNDTFGPAENALHSDPHIEGDHMSEFAVSDKPKLPNDALCRSYQALEVEPSEVLVAAETAGDEYSRPREDKIIIRQTYKKEVVLSKKSPICPGRPPKATLLANIDAMEAGGRTGKRENPAATNPRYLNLEPSLAMDWLEISWDELQMKERVGAGDVAVKLLTVQDFHDDQLKEFLREVAIMKRVRHPNVVLFMGAVTKRPHLSIVTEYLPRGSLYRLIHRPAAGELLDQRRRIRMALDVAKGINYLHCLNPPVVHWDLKSPNLLVDRNWNVKVCDFGLSRFKANTFISSKSVAGTPEWMAPEFLRGEPSNEKSDVYSFGVILWELVTMEQPWNGLGPAQV

>NtEDR1-17

MEEIPDEVELPEHRYPNTAWWPSDYVEKLGSVSLDGKEEIMRNREPTEREYDRLSSQTASQILWKTGTLSEPIPNGFYSVVPEKRLKELFEDIPTLDELHALEPEGLGADVIVVDTKKDKKLSMLKQLIVALVKGLNSTPAAMIKKIAGLVGIHISFSLNLHINLFHEFMIFLKNRGVRANLCAPRLIPATPTSSSSILSHSEPNTASTVWRRGRRKVITEPRTASSSPEHPSFRAHGRSMLSGDNKTFRDYSDDVATSRSEGASTSETRRLRRRSISITPEIGDDIVRAVRAMNEALKQNRLLKEQEENTSFHASNNRDCASDLKKDISSQKAMSLPSSPHELRRQAPESSGPDRTNNELFSTWNRILESHMYQNKPLLPFEEWNIDFSELTVGTRVGIGFFGEVFRGKWNGTEVAIKVFLEQDLTAENMEDFCNEISILSRLRHPNVILFLGACANPPRLSMVTEYMEMGSLYYLTHLSGQKKRLSWQKRLNMLRDICRGLMCLHRMKIVHRDLKSANCLVNKRWTVKICDFGLSRIMTDASMKDLASAGTPEWMAPELIRNEPFTEKCDIFSFGVIMWELCTLNRPWEGIPPDRVVYAVANEGARLEIPEGPLGRLIADCWAEPNERPSCEEILTRLLDCEYSLC

>NtEDR1-18

MKNLLKKLHLMPNESVDSEGSTSSAKTKRLSDVSSPDNNPFSAISGWLNSVTNRHNSPSPQSSSNMSRGNNNRMEPCDSASSSGLDAALDTVRRDSESSNSREPDIEEEYQIQMALELSAREDPEAVQIEAVKQISLGSCAPENTPAEIVAYRYWNYNALSFDDEILDGFYDLYGIMTESNSSKMPSLVNLQRTPVSDQITWEAILVNRAADSKLLKLEQKALEMSVKVRSESIDFTGGNLVQTLAMLVSEHMGGRVGDPDSMLVAWRSLSYNLKATFGSMVLPLGSLTIGLARHRALLFKVLADSVGLPCRLVKGQEYTGSDEVAMNYVKIEDGREYIVDLMADPGTLIPSDTSGTQVDCEESILSISPSSKDVDSHMGGSSSSGVACSSEDHSEYGTAERRSRFRESSTGNESPSSSNNSEKQIKAEKGCNNSSNEFTKLWTVKKEQRQETSPGTGHARSSYTHARSPSWTEGVSSPAVRRMKVTDVSQYMIDAAKENPQLAQKLHDVLLESGVVAPPNLFTEVYTEELDVSPVEGKSGPEDMESKGSDEIEKNKSQADLDRNSFLPPLPYHVMSKGNPRGPFDSGQQVSPHSELAAVKFTKNMPVAAAAAVAAAVVASSMVVAAAKTTYGSKADLPVAAAVTATAAAVVATTAAVTKQYENLEPSSDSPDSPAFFLNLIDSGRVDGDADGAVYEQRGSGDQVHEALGVNSENERISDRSTGNDSVKSDVTLDDVSDCEIPFEDITLGERIGLGSYGEVYRGEWHGTEVAVKKFLDQDITGESLEEFKSEVWIMKRLRHPNVVLFMGAVTRPPNLSIITEFLHRGSLYRLIHRPNNQLDERRRLRMALDTVCDFGLSRMKHSTFLSSRSTAGTVSRRDLIQLLGTCFFQP
