# Supplementary material for: Identification and expression analysis of EDR1-like genes in tobacco (Nicotiana tabacum) in response to Golovinomyces orontii
Source: PeerJ. 2018 Jul 10;6:e5244. doi: 10.7717/peerj.5244 (PMC6044316; doi:10.7717/peerj.5244)
Supplement: Supplemental Information 6 [file peerj-06-5244-s006.docx]

>SlEDR1-1

MKHIFKKLHHSNRSNDAQSTSSISSSSSPASSLSSASCTTDHRNSNSVSQSPLSPSTISTASTTTTPAAPVGAGGGGGGGNLSTINRQQDYYTSEEEYQVQLALALSVSSSQSQDPFPSDVNSSNGHGVGRTAVDLARDREDAAADLLSRQYWDYGVMDYEEKVVDGFYDVYNLFTDPASRGKMPSLSELETNPGTSNFEGVIINQRIDPSLEELMQIAHCITLDCPASEISLLVLRLSELVTGHLGGPVKDANIILAKWMEISTELRTSLHTSVLPIGSLKIGLSRHRALLFKVLADHVGIPCRLVKGSHYTGVEDDAVNIVKLPNDSEFLVDLMGAPGTLIPADVLSAKDASFNSPKLNKIPSLPSNSHSGVSYPRRNLLSGQNSVLGDDFSGRSKPEKIESVHSISDAGGSSTAGSSGINKRPSSNQVDWTSPLAIGTSLYKGGRGPNAAGDGLRLNVNVVPYDQNNPEDPKNLFADLNPFQIKGSGNTLLQKNPARNKVSELQQPINTLIPGRPPAPMMWKNRYAPNEVPRKNESDSEGLFPKKNGGSSGYNISSIASTSSNIPQKSSTDTSRLHGNSRPAYRGNDEVASTRNNSSILSAELEFRRLSVQNSQNNNRETSQWEGHSLQSDDLNRTQAYGDDIIVESDHTRNLQAQSIGTNIKLKEPENPTSSGNLGPSQVDPVFDDVGDCEIPWEDLVIGERIGLGSYGEVYHADWNGTEVAVKKFLDQDFSGAALAEFKREVRIMRRLRHPNVVRFMGAITRPPHLSIITEFLPRGSLYRIIHRPHFQIDERQRIKMALDVAKGMDCLHTSNPTIVHRDLKSPNLLVDTDWNVKVCDFGLSRLKHNTFLSSKSTAGTPEWMAPEVLRNEPSNEKCDIYSFGVILWELATLRLPWSGMNPMQVVGAVGFQNKRLEIPKELDPIVARIIWECWQTDPNLRPSFAQLTVALTPLQRLVIPAYVDQLNSRLPQEISVNSTP

>SlEDR1-2

MFASGKSCGVPFTSLLDEQNYNVVNYDEKVMDGFYDVYGINPCAVIQGKMPLLVDLKAVSVLDNVAYEVILVNRAADMELRQLEERVYFMSRECRALKKVPVTSFLVEKIADLVVNRMGGLVNDAEEMSKRWTARSYELRISLNSIILPLGCLDIGHSRHRALLFKVLADRINLPCKLVKGSYYTGTDDGAVNLIKFDNGSEYIIDLMGAPGALIPTEAPTGQLQSYAVDVHSVTPLPSGGTVISFPVFDTQTRTGSGSVNAAHGTANTWISREEPAFYHNEAKGNYGNSSGRTGSTQFEHDSGNLPPLSARLCDASAVSHDNASIAQITQAREAYENVNSLAENSEAKLLGVSPESQMYLQSDLVLGVVAGKNQLSEERAVNTRQSSENNNQSLVTFTGMQFPYSISYESEQEYTVALPRNDTLNDTSGDKFFRGEFGNISHNDCTYKDKESATKAREIVTCIQSKSYAVQKEQLDPMLRGVAEWEIPWEDLHVGERIGIGSYGEVYRAEWNGTEVAVKKFMNQDITSDALEQFKCEIEIMLRLRHPNVVLFMGAVTRPPNLSILTEFLPRGGLYKLLHRPNILIEEKKRMRMALDVAKGMNYLHTSNPIIVHRDLKTPNLLVDKNWVVKVCDFGMSRMKHHTFLSSKSTAGTAEWMAPEVLRNEPSNEKSDVYSFGVILWELTTLQVPWTGMNSMQVVGAVGFQGRRLDIPPSVDPIVAEIISECWNQDPQVRPSFAQIISRLKRLQRLNIQGFETCTNRH

>SlEDR1-3

MSGRRSSYTLLNQIPNDNFFQPPAPKFSAGAGVAPYGESSSAEKNRGKVFDLDLMDQRMMQSHNRVGSFRVPGSIGSQRQSSEGSFGGSSLSGENYVGTSFGHKNEGCGSSVARSWAQQTEESYQLQLALAIRLSSEATCADSPNFLDPVTDVLASRDSDSTASAVTMSHRLWINGCMSYFDKVPDGFYWIYGMDPYVWALCSVVQESGRIPSIESLRAVDPSKAPSVEVILIDRCNDLSLKELQNRIHSISPSCITTKEAVDQLAKLVCDHMGGAAPAGEEELVSMSKGCSNDLKDRFGTIVLPIGSLSVGLCRHRALLFKVLADIIDLPCRIAKGCKYCNSSDASSCLVRFEHDREYLVDLIGKPGVLSEPDSLLNGPSSISIPSPLRFPRYRQVEPTTDFRSLAKQYFLDSQSLNLLFDDSSAGAAADGDAGQSDRSCIDRNNVVSSSSNRDEISQLPLPPLNAWKKGRDKESQLSKMYNPRSMLNPVNMDEDQVLVKHVPPFREDAQSPMTRPDTVNDTRFLAGGGHVVSAIPSEELDLDVEEFNIPWNDLVLMEKIGAGSFGTVHRGDWHGSDVAVKILMEQDFHAERLKEFLREVAIMKRLRHPNIVLFMGAVIQPPNLSIVTEYLSRGSLYRLLHKPGAREVLDERRRLCMAYDVANGMNYLHKRNPPIVHRDLKSPNLLVDKKYTVKICDFGLSRFKANTFLSSKTAAGTPEWMAPEVIRDEPSNEKSDVYSFGVILWELATLQQPWNKLNPPQVIAAVGFNRKRLDIPSDLNPQVAIIIEACWANEPWKRPSFSTIMDMLRPHLKSPLPPPGHTDMQLLS

>SlEDR1-4

MEVPVRRSNYAILQQQPPYDEFNSDEKSKSRGDKGLYWDLIDRRKGTTPFQASIVLPTQSSEGSFAESSISGVSFGYMNAYSDVGGSLSKSWAQQTEESYQLQLTLALRISTEATCADDPNLLDYVPDESVSHASASSASVEAMSHRFWVNGSLSYFDKVPDGFYFIQGMDPYIWTVCSDLQESGRIPSIESLMAVDPSVVPSVEVILIDRQSDPRLKELQNRIHSMYRSCNTTKEVVDQLAKLVCNHMGGAASVGEGDFIPIWKECCNDLKDCLGCFVFPIGSLSVGLCRHRTLLFKVLADIIDLPCRIARGCKYCKESDAFSCLVRFGLDREYLVDLIRDPGCLYEPNSLLNGPSSISIPSPLRLPRFGQVEPAMDFTSFAKQYFSDCLSLNLAFDDSSAGTAVDGDAGQTDRSSMDKSSAVPSSSNRDEVSRLPLPSINAWNKGCDKGSQLPAKYHPPNMSISMSQEKDLIHLKNVPPIRYVDAHLIAISEARTDTINDQRYFEGVGRLAPAKPSRGLVLDVEDLDIPWNDLVLKERIGAGSFGTVHRADWNGSDVAVKILMEQDFHAERYKEFLQEVAIMKRLRHPNIVLFMGAVTEPPNLSIVTEYLSRGSLYRLLHKPGAREVLDEKRRLCMAYDVAKGMNYLHKRKPPVVHRDLKSPNLLVDTKYTVKVCDFGLSRLKANTFLSSKSAAGTPEWMAPEVLRDEPSNEKSDIYSFGVILWELATLQQPWSNLNPPQVVAAVGFKGMRLEIPRDLNHPVTTIIEACWVNEPWKRPSFSTIMDMLKPLI

>SlEDR1-5

MEMSTRRSNYTLLSQVADDNYLPPPPKYSVTGGGGGGGGVAPYYESHSGEKGKGKTGDNRGFDWDLSDHRSNMMQASNRIGAAAFPGSIGLQRQSSGSSFGESSISGEYYMPSLSNAEASFGYLNDGGGGAEVRMKPLEANLFGGSSSKSWAQQTEESYQLQLALALRLSSEATCADDPNFLDHVPDESASRASASAASAETLSHRFWVNGCLSYFDKVPDGFYLIHGMDPYVWIVCSDLQENARVPSIESMRAVDPSVVPSVEVILIDRRTDPSLKELQNRIHSLSPTCGTTKEVVDQLAQLVCSHMGGATSAGEDELVPLWKECSYELKDCLGSTVLPIGSLSVGLCRHRALLFKVLADAIGLPCRIAKGCKYCNRADASSCLVRFGPDREYLVDLIGSPGCLCEPDSSLNGPSSISISSPLRFPRFREVEPTTDFRSLAKQYFSDCQSLNLVFEESSAGAAVDGDAGQTDRNNIERNSAVTGPSNRDEVSRLPVPAIRDMAPVKYVRPVLHGDTQLSDPRDIGNDMRFLERGSQLVPSKISRDIALEIEDFDIPWEDLVLKERIGAGSFGTVHRADWNGSDVAVKILMEQDFHAERFKEFLREVAIMKRLRHPNIVLFMGAVTQRPNLSIVTEYLSRGSLYRLLHKPGAREVLDERCRLSMAYDVAKGMNYLHKRNPPIVHRDLKSPNLLVDKKYTVKVCDFGLSRLKANTFLSSKSAAGTPEWMAPEVLRDEPSNEKSDVYSFGVILWELATLQQPWSNLNPAQVVAAVSFKGKRLDIPRDLTPQVASIIEACWAKEPWKRPSFAAIMDMLRPLIKPPVTPPQPGRTDTQLIA

>SlEDR1-6

MPHRTTYFFPRQFPDRGLDASAKFVNDHEKEKEKKISDVEDRKSSSKERDVVASKQLISDVKETDNNNDDATFSYGNRDKIHGKQLAAFVNWLTEKNKKGKSIRNHVKIKLDDGDTEDEHELLLPVPPEAVPIHELQVDCHVAPLEQKQQGTFDRKASLQRLSSSGSNYSCVGKQFERQTSLQRLSSWGSTSYAGSLFSGTTVDGNWPSTGVKDTQTSTTREVEEEVVGQDAEERVDNEDTLIQKSKESYYLQLTLAKKLVEQAMLASGEPILLQECKNIKGLGGSSDAQTVSYRLWVSGSLSYADKISDGFYNILGMNPYLWVMCNATEDGKQIPSLMALKGIEPSETSMEVVLIDRRGDSMLRELEDKAQEIYFAAENTLVLAENLGKLVAVYMGGSFPVEQGDLHQRWQAVSKRLQDLQKCIVLPIGSFSSGLCRHRAILFKKLADYVGLPCRIARGCKYCVADHRSSCLVIIEDDRRLSREFVVDLVGDPGNVHGPDSSINGGVLSRVPSPLQVSHLTEFQQPYMDSDISNQLLHSNDTFAAPENALHTDPHVESKHVKGIVVSDKPKFPNDPLYQPYQALEAKPCEVLVAAETAGDENSRPREDKIIIRQTYKKEVVLSKNSPLQSGRPPKSTLIGKMDVMEPGGRTGNREKHPTTTNPRYLHLEPFLAMDWLEISWDELHIKERVGAGSFGTVHRAEWNGSDVAVKLLTVQDFHDDQLKEFLREVAIMKRVRHPNVVLFMGAVTKRPHLSIVTEYLPRGSLYRLIHRPAAGELLDQRRRIRMALDVAKGINYLHCLSPPIVHWDLKSPNLLVDKNWNVKVCDFGLSRFKANTFISSKSVAGTPEWMAPEFLRGEPSNEKSDVYSFGVILWELVTMQQPWNGLSPAQVVGAVAFQNRRLTVPQNTSPMLASLMEACWNDDPVQRPSFASIVDTLKKLLKSPLQLIQMGGTMKS

>SlEDR1-7

MEERGDEVGPSEQRSPGAACWPSDFVEKFGSVSLDSKEENLRNKEPSENEIHDRLPCQTASQILWKTGTLSDPIPNGFYSVVAEKRLKEIFEDIPTFDELQALELEGLRADIILVDFEKDKKLSMLKQLIVALVKGLGSNPAAIIKKIAGLVSDVFKRSNPELSPSKAALEESSHISEIRGIQMLGQIKHGSCRSRAILFKVLADTVGVESQLVVGLPADGASECVDSSKHMSVIVVLNSVELLVDLMRFPGQLIPRSAKAIFMTHISAAGESDSAENDSCDSPLEPNSPLYGFSERIDPESSEKDDALQCQRRLEASSNAAGPSLRNMMLRSTSIDRKLSFSHSEPNIATAAWRRSRRKVITEQRTASSSPEHPSFRARARSMLSGDTKTFKDYSDDVATSRSEGASTSEPRRLRRRSISITPEIGDDIVRAVRAMNEAMKQNRREQGENSSSPHSSNDRGGTLDLQKNVSDFHHDDPEISRGQSSMFPLSREHLSSQKAVSLPSSPHEFRRQALEGRGQMNDKMVSTWNKILESPMFVNKPLLPFEEWNIDFSELTVGTRIGIGFFGEVFRGIWNGTDVAIKVFLEQDLTEENMEDFCNEISILSRLRHPNGISLIFFFSRIGFSFLLGSIILLLPSTVAVILFLGACTTPPHLSMVTEFMEMGSLYFLIHLSGQKKRVNWRRRLKMLRDICRGLMCMHRMKIVHRDLKSANCLVNKHWSVKICDFGLSKIMTDAPMRDTAAAGTPEWMAPELIRNEPYTEKCDIFSLGVIMWELCTLERPWHGVPPERVVYIVANEGSRLEIPEGPLGQLITDCWAEPKDRPSCAEILTRLLECEYAIC

>SlEDR1-8

MEKTGNDTGPSEALPPDSPWWPADISDKLQLITLVSSEDKANSTVLSSKQEREGLASKRASQILWDTGELAEPIPDGFYFVIPERRFKELFDTIPSLDDLYALETEGLRPNVILVNMHKDKKLSMLKQLTLTLVKGLSSTPAVVVKKIAGLVCDFYKHPKYKSTHVSGTLEEVSHALGSQGIHMLCQIKDGSCHSRAILFKVLADTVGLECKLIVGFPRGGALECADSSKHIYVTVILDSIELLVDLMHCPGKLFPCSTKQLHRSHFFGESDSVETDSCNSPMGPISPTCFSSDYSGTGSPEHPFLRGPGRSILGGRAQSFKDCNYDVTSSRSAGASPIHTRRRRRRSISMIPEIGDDIVRVVREMNATMKKNHLSAEQATFDLSTRKGGDNFGHQVQNFRSDDLNTGNSEKGQAPHFHRKCLPSDKAISLPSSPRWSYGRGKAAGIFGSPDMMSRLDKVIESSRILNKPLLPFDEWNIDFSEITIGARVGIGFFGEVFRGIWNGTEVAVKVFLEQELTEENIEDFANEISILSRIRHPNVILFLGACTTPPRLSVVTEFMEMGSLYHLIHVSGQKNNLSWQRRLKMICDICRGLMCIHRMKIVHRDLKSGNCLVNKHSKVKICDFGLSRSLTPSPMQDSSSAGTPEWMAPELIRNEPFTEKCDIFSLGVIIWELYTLKRPWEGVPPIQVVYAVANDGKRLEIPEGPLGKLIADCWAEPDERPSCEEILSRLGECIRSSAN
