# Supplementary material for: Identification and expression analysis of EDR1-like genes in tobacco (Nicotiana tabacum) in response to Golovinomyces orontii
Source: PeerJ. 2018 Jul 10;6:e5244. doi: 10.7717/peerj.5244 (PMC6044316; doi:10.7717/peerj.5244)
Supplement: Supplemental Information 7 [file peerj-06-5244-s007.docx]

>OsEDR1-1

MKNLFKSKIKWQHRSNDPASSQGQGQGLPQQTPPSPSPASSPSGGPPALSVSTVSSSSPSAAATPTGAAAAGAGGGGGGTGGEDYMLSEEEFQMQLAMALSASNSECVGDLDGEQIRKAKLISLGRGDRFAAVRDDEQTADALSRRYRDYNFLDYHEKVIDGFYDIFGPSMESSKQGKMPSLADLQTGIGDLGFEVIVINRAIDTTLQEMEQVAQCILLDFPVANIAALVQRIAELVTDHMGGPVKDANDMLTRWLEKSTELRTSLHTSLLPIGCIKIGLSRHRALLFKILADSVGIPCKLVKGSNYTGDDDDAINIIKMNEREFLVDLMAAPGTLIPSDVLSWKGNSLNSNARLTQNPLAGSSSTTDSNLSANALPPGHKGGQLPLFSSGDWISASQSGYEKDGATTSSQASSSGTTSVAAGSAFDSSWTLVSHGQSDDPSTSAGMSAQQKVILPGGEHPWNENINARNENIKLVSDLQGNSESINLFADLNPFGGREPKRTSVPLNGPDNRNNELQRRRENVVPSTRRPQQRLVMKNWSPYNDVSNNKQYNYVEDSFARRNIGDNAASSSQVPRPSAKNTNLNVVVRTDTPYMAAHNYDNSMAGSSAMKMTSTAGIGKVPDKVLYGDLDKGLTNSRLGDQPPIERHKWGNSVEGRIPTGTVHNQAKEHKENFDGKQDNKKLHPDPKKSPLDRFMDTSMPSRNPESVSPSFARSHKLDTMFDDVSECEIHWEDLVIGERIGLGSYGEVYRADWNGTEVAVKKFLDQDFYGDALDEFRSEVRIMRRLRHPNIVLFMGAVTRPPNLSIVSEYLPRGSLYKILHRPNCQIDEKRRIKMALDVAKGMNCLHISVPTIVHRDLKSPNLLVDNNWNVKVCDFGLSRLKHSTFLSSKSTAGTPEWMAPEVLRNEQSNEKCDVYSFGVILWELATLRMPWSGMNPMQVVGAVGFQDKRLDIPKEIDPLVARIIWECWQKDPNLRPSFAQLTSALKTVQRLVTPSHQESQSPPVPQEIWVNSSTP

>OsEDR1-2

MKNFLRKLHIGDSAGDGASSLAPPPPVSKKGGGGGGGGGGGGAQHEHKHGSGISSWLSSVTGRPQTQPSPSPPFAADAVVEAEAAALASSVEVRRLEVEEEEEKARRESREESVRKREMEKEKQEAELEEYHMQLALEMSAREDPEATQIEVAKQISLGSCPLQSSPAEVVAFRYWSFSALSYDDKILDGFYDIFVIGDEPTLPTIPSLTELHQQPFSHASKTEAVLVNRAQDTKLVQLEQKALIMAVEVRSKTPEFVGHNLVQRLATLVSDYMGGPVIDPESFLSKYQNVSSSLRASIRSAVMPLGELTIGLARHRALLFKVLADSLAVPCRLVKGRQYTGSDDGALSIVKFNDGREYIVDLMSDPGTLIPSDGAGLGREFEDSLFADSHHVNKDDCNTQLGSSFSEVSSSMYGSFENESLEKVSTPSNFGHSDPYGITTGQTGSQGSAVSGSFGELSISTSTSENLPVIHESRNTDHTMSTQSKDKSSAANNSSSSSPSSSEVGGAPAVRRMKVKDVSEYMISAAKENPQIAERIHAVLLENGVVPPPDLFSEESREQPKDLIVYDTSLFQTKDEMIKRMNELESTTNADFCHGPSVPHPPGHELQTKAVPYRIPLDLKPIQGLGTYHPSDSRNSTGSSHMYEPSAPPQEDPLQLIKQMPVAAAAVATAAVVASSMVVAAAKSNSDIKLDVPVAAAATAAAVVATTAAVNKQYEYLEPGCQLLSLPSSSGANELIPKGRHDFWDNQLEIDHGQTSVPEKEKDLVEVPQEAERVSDKSVGTESSRSDIALDGVAEFEIQWEEITLGERVGLGSFGEVYKGEWHGTEVAVKKFLQQDISSDALDEFRTEFQIMKRLRHPNVVLFMGAVTRVPNLSIVTEFLPRGSLFRLIHRPNNQLDERRRLRMALDVARGMNYLHNCSPVVVHRDLKSPNLLVDKNWVVKVCDFGLSRMKNSTFLSSRSTAGTAEWMAPEVLRNEPSDEKCDVFSYGVILWELFTLLQPWEGMNPMQVVGAVGFQQRRLDIPAHVDPTIAEIIRRCWQTDPKMRPSFSEIMSSLKPLLKNTLANQPQRQRVQRADG

>OsEDR1-3

MKNFFRKLHIGEGSGDGASSSPPPPPSSRKGSGGVGGNHHLHAEQRQPSASAVSSWLDSVPGRPQPPTPSTPSEAEGSPFSSSVGSGAEERRQSVAAERRRSQEEEWERRRSQEEEAVREMRRSQEEDEVEERVIRESSEAEERKRVREKEDDDLEEFQLQLVLEMSARDNPEEMEIEVAKQISLGFCPPQSSTAEALAARYWNFNALGYDDRISDGFYDLYVTGNGPASITMPSLKDLRAQSLSHRVNWEAVLVHRGEDPELMKLDQTALIMSLELRESKPSEFVGNDLVQKLAGLVARHMGGTFFDSEGMLVKYQKMMRYLRTSIGSVVVPLGQLKIGLARHRALLFKVLADNIGIPCRLLKGRQYTGSDDGALNIVKFDDGREFIVDLVADPGTLIPSDGAVLSTEFEESSFSNNHHFNKDNDIRQLGSSNSLSNSACSSFECELLDRRSTWINVGPSDSDGATTSQTSKNNQQNTLSDSFGILSVSTFTSENRPITNESRSTDDIAAAKNKERSSVTINSSSTSPSPSSPEVGSTPAVRRMKVKDISEYMINAAKENPQLAQKIHEVLLENGVVAPPDLFSEDSMEEPKDLIVYDTTLFQSKDEMKKRMNELGSREYADRGHGPLLPHHPGHELPSKVPHRAPLDSLKPVEGLGIDHPPDIQDNTSFISQYEPSAPPQEASSQLTKQLPVTAAAVATAAVVASSMVVAAAKSNNDVNFDVPVAAAATVTAAAVVATTAAVSKQYEHLEPGNQLHSLPSPSEGNESIEKSADEFWDKQNFEIDHGQDNTLDQEKDSAEVRQDAERTSDKSSGTESAKSEITLDDVAEFEIQWEEITIGERIGLGSFGEVYRGEWHGTEVAVKKFLQQDISSDALEEFRTEVRIIKRLRHPNVVLFMGAITRVPNLSIVTEFLPRGSLFRLIHRPNNQLDERKRLRMALDVARGMNYLHNCTPVIVHRDLKSPNLLVDKNWVVKVCDFGLSKMKNKTFLSSRSTAGTAEWMAPEVLRNEPSDEKCDVFSYGVILWELCTLLQPWEGMNAMQVVGAVGFQNRRLDIPDNTDPAIAEIIAKCWQTDPKLRPSFADIMASLKPLLKNMTAQAPRQRVQQTDE

>OsEDR1-4

MKIPFVTKWSHRSHEPAAPSNPAAQQQPPPPSPAGVSSAAAAAVVEEAEMETGGDDFITQEEEYQIQLAMALSASASVSAPSGGGGSGDTEGEQIRKAKLMSLGRGDLSAAADRGVGDSAEALSRRYRDYNFLDYNEKVIDGFYDIFGLSGESARQGKMPSLAELQTSIGDLGFEVIVVDHKFDSALQEMMEVAQCCMLGCPDTTVLVRRIAEVVAGHMGGPVIDATEMFTKWLGKSIEQRTSHQTSLLPIGRIDIGLSRHRALLFKILADSVGIPCKLVKGSHYTGVEDDAINIVKMNNEREFLVDVMAAPGTLIPADVFISKGTPFNLTKPLVQNQVVELASNIENDPSAAHSEHVGNRLHMFGNGNSLSENQSGCEKTMIAGSEVSQLWTLAPQMQSDQQSTSAGAHSMQKEDLKLTPDSQENEESKKQISETDSFRGIELGKSSLAFKGLNNRNNEYQRHRENIAPAPGRSQQPLVMKNWSACNDISNKQYNIAERLVRRRNTSDNAASSSQLAWSTAKHYNPNGRERNDRLCAAPGRNYDNRKVGASTTATASATGERLDRPNLAPVHYYDDKPNGISSVNAASTSGIVKVAEKGPHDLEKVPIYSRFDSQIYSSMQGYSPEVKENKENYDRHDNMRLHPDPRRSPLDRFMDTSRQNSESVSPPQAGSSTVDMVLGEVSECEILWEDLLIGERIGLGSYGEVYHADWNGTEVAVKKFLDQEFYGDALAEFRCEVRIMRRLRHPNIVLFMGAVTRPPHLSIVSEYLPRGSLYTIIHRPDCQIDEKCRIKMALDVARGMNCLHTSVPTIVHRDLKSPNLLVDNNYIYRSVILDFHV

>OsEDR1-5

MKHLLRKLHLSGGGGSGGGAAAAGAPSGEHHHRPRQHRRSSAQPPPLPPPPVVAAAAAAEAAPVMAPVAAPVAAAAAEPRGMGADATMTRLEEEYQVRLALAISASDHAGLVDADSVQIRAAERISLGGAAGDRGPMEALSARYWNHCVVNYDERLSDGFYDVCGAPMHPHFQAKFPSLTTLRAVPVGGDAAYVAVLVNRERDPALKRLEGRALAIAAQDRAEHGGVASPELVQKIANLVVDAMGGPVDDADEMNREWGVKSRALCLQRNSIVLPLGLLRIGLSRHRSLLFKVLADRVNLPCKLVKGIYYTGTDEGAINLVKIDFDSVEYIVDLMGAPGTLIPSDISGSQFQDSNNSQLSNDAIEESVAELCIALEQISAGCKNTSDMGGSSSEQKSALALASSQLEDIFHTENPLKQSTISDGGEIPHLMKVNDAPMYLVPTEVDPQFAQNLQDLLLEGTALLPTYEKPEICKHTASEDDKTAGWLVIAKTGQNLPNGHVAEDSPLQHGNTKTLAVVNCFHEDAQHDVGNTEAIGRNLDLHDHTAHAIANEDQRFSEDSLVKMPGSSNGNLDKSSCSSTKTISSVIDDVADYEIPWEDLHIGERIGLGSYGEVYHADWNGTEVAVKKFLDQDLSGVALDQFKCEVGIMSRLRHPNVVLFLGYVTQPPNLSILTEYLPRGSLYRLLHRPNSQIDETRRLKMALDVAKGMNYLHASHPTIVHRDLKSPNLLVDKNWVVKVSDFGMSRLKHHTFLSSKSTAGTPEWMAPEVLRNEPSNEKCDVYSFGVILWELATMRVPWSGLNPMQVVGAVGFQNRRLEIPKEIDPLVATIISSCWENDPSKRPSFSQLLSPLKQLQRLVVPENC

>OsEDR1-6

MKADAKWPAMVLGGGGGGGRRASPGSAPPPAAPAAVAYSLLATSPPASIGNGGGSPHCDDGDASRGLGVADWLRLQRHSSGSSAGDDGDGFSSVSTLATADKGGDPADRPAGSSGGGGSKSWAQQAEEAYQLQLALALRLCSEASTAPDPNFLDSAVAAADDHHRDAPSPQSLSHRFWVNGSLSYSDKVLDGFYLIHGMDPFVWTLCNDLRDGARVPSIESLKAMNPTESSVEVVLIDRVVDYDLRQLISTAIDVSRSRADSREITTRLAGIVSSKMGGSVASTEEHELCPRWRDSAGFLKISSGSVVLPIGKLSIGLCRHRSLLFKTLADTISLPCRVVRGCRYCKSAGAASCLVHFGNDREYLIDLIGNPGFLSEPDSLLNGLSSISVSSPLRPPKYNSADIVNNFKSLAKQYFLDCQSLNMMFNDPAAGTVVDLDEAMGSNIGPNLSPATNSDFQANFSHRSRGAQSSGQDGNFLIQKSSPEDTQSAQSDPFSDISLDIEDLIIPWSELVLKEKIGAGSFGTVHRADWNGSDVAVKILMEQDFHPERLKEFLREVAIMKSLRHPNIVLFMGAVTQPPKLSIVTEYLSRGSLYRILHKHGARENLDEKRRLSMAFDVAKGMNYLHKRNPPIVHRDLKSPNLLVDKKYTVKVCDFGLSRLKANTFLSSKTAAGTPEWMAPEVIRDEPSNEKSDVYSFGVILWELMTLQQPWSTLNPAQVVAAVGFNGRRLEIPSSVDPKVAAIMESCWTKEPWRRPSFASIMESLKPLIRTPHQLQEDIS

>OsEDR1-7

MKADAKWPAMVLGGGGGGGRRASPGSAPPPAAPAAVAYSLLATSPPASIGNGGGSPHCDDGDASRGLGVADWLRLQRHSSGSSAGDDGDGFSSVSTLATADKGGDPADRPAGSSGGGGSKSWAQQAEEAYQLQLALALRLCSEASTAPDPNFLDSAVAAADDHHRDAPSPQSLSHRFWVNGSLSYSDKVLDGFYLIHGMDPFVWTLCNDLRDGARVPSIESLKAMNPTESSVEVVLIDRVVDYDLRQLISTAIDVSRSRADSREITTRLAGIVSSKMGGSVASTEEHELCPRWRDSAGFLKISSGSVVLPIGKLSIGLCRHRSLLFKTLADTISLPCRVVRGCRYCKSAGAASCLVHFGNDREYLIDLIGNPGFLSEPDSLLNGLSSISVSSPLRPPKYNSADIVNNFKSLAKQYFLDCQSLNMMFNDPAAGTVVDLDEAMGSNIGPNLSPATNSDFQANFSHRSRGAQSSGQDGNFLIQKSSPEDTQSAQSDPFSDISLDIEDLIIPWSELVLKEKIGAGSFGTVHRADWNGSDVAVKILMEQDFHPERLKEFLREVAIMKSLRHPNIVLFMGAVTQPPKLSIVTEYLSRGSLYRILHKHGARENLDEKRRLSMAFDVAKGMNYLHKRNPPIVHRDLKSPNLLVDKKYTVKVCDFGLSRLKANTFLSSKTAAGTPEWMAPEVIRDEPSNEKSDVYSFGVILWELMTLQQPWSTLNPAQVVAAVGFNGRRLEIPSSVDPKVAAIMESCWTKEPWRRPSFASIMESLKPLIRTPHQLQEDIS

>OsEDR1-8

MPHRRRLLNPAPSLPPPPAAAPAGFHHHLLAVDDTRLPLLADYALLQGDAAAAPASAEWSAGSGFTGISTDPATATTATTASTATAPGSASNLTAATAGGGGRETWVRRAREGYYLQLSLAIRLTSEAFLAGVPPELLIGCGGGGEAENHADVAADAAAVSYRLWVNGCLSWGDKIAHGFYNILGVDPHVWAMCNAAAEDGRRLPTLVALRAVDSGESSVLEVVLVDKCGDPALADLERRALDLYRAAGVSLDLVRHLAVLVSDHMGGALRSEDGDLFMRWKAVSKQLRKRHRCVVVPIGSLSIGFCRHRAILFKSLADFIGLPCRIAQGCKYCSAPHRSSCLVKIDNERRFVREYVVDLVVEPGRLSSPDSSINGQLLSSVPSPFKTSCTMSSANYATPAASWNRAISGDRRNSILSNPQYSVAKYCVAEEKSSVQVATKEAMLPKCGQITQNGNCNKNSMAVFEVSKQMKAMEISSESGDKDNISSATPLKRLSIEPSFCADWLEISWDEIELKERVGAGSFGTVYRADWHGSDVAVKVLTDQDVGEAQLKEFLREIAIMKRVRHPNVVLFMGAVTKCPHLSIVTEYLPRGSLFRLINKASAGEMLDLRRRLRMALDVAKGINYLHCLNPPIVHWDLKTPNMLVDKNWSVKVGDFGLSRFKANTFISSKSVAGTPEWMAPEFLRGEPSNEKCDVYSFGVILWELMTMQQPWNGLSPAQVVGAVAFQNRRLPIPQETVPELAALVESCWDDDPRQRPSFSSIVDTLKKLLKSMLGGS

>OsEDR1-9

MDLPAVTGRRTTSYSLLSQFPDDAAVLQRQSSGSSYGAGSSLSASSDFPFHLPSAAAPAAGAPGGSPCKSWAQQAEETYQLQLALALRLCADAASAADPAFLDPGHSATATAGPFPLPPPTPSADSLSHRFWVNGSLSYSNTIPDGFYLIHGMDPFVWSLCTDLLEENRIPSIDSLKSVRPDDSSMQAILIDRRTDFDLGMLENYASSFLSSSADMKDVINQLAKLVSSRMGGTTSNEESFLPRWKECSDAIKSSTGSIVLHLGKLPIGFCKHRSLLFKMLADKVNVPCRVVKGCKYCKSDDATSCLVRFGLEREYLVDLIGDPGQLSDPDSFVNGPYSLSVPSPLRPPKFRSLEITSNFSSVAKQYFSDCHSLNLLFNEASTGANSNAAVAMDQPYSTRKHDTRDDIMSSWVPVKGQAAVSSDAILPEAPREVLPLITSSNLKAEKKKEFKLIEGNQYLRSTVSDLSLAVDDLIIPWNELILKEKIGAGSFGTVHRADWNGSDVAVKILMEQDFHPDRFREFMREVAIMKSLRHPNIVLFMGAVTEPPNLSIVTEYLSRGSLYKLLHRSGAKEVLDERRRLNMAFDVAKGMNYLHKRSPPIVHRDLKSPNLLVDKKYTVKVCDFGLSRLKANTFLSSKSLAGTPEWMAPEVLRDEPSNEKSDVYSFGVILWELMTMQQPWCNLNPAQVVAAVGFKGRRLDIPKDLNPQVAALIESCWAKIILSGYRLLAKLSSVQNGLFVVTEMRVRARSGRRWSSWVLLDYVAFISNSNHHGNATTARSKTRTGQPIEVSFWTAPPPRVSYMCVHCPRLDPYKFATEPTIMATEADLVLIRVTIGPRGNCFNTKCSDLFIFFDREVGLLRCGSVRRRHDPTRLGLHAHTGPDDGTYIVAVLCNTFQSGNLEYALYLYRAGADAWTCHPLSLHGLVDPTSFIHVNTNTITVGGEAGTMAWVDLNRGILFCDLLPCPYTPLLLRYFPLPPPLRLSAHTKLTGCPRFSHDIALVQGRFNFTQMRIHVKPGSITNGTYISQGWTLATWSAPATNPWKQGWRQDCNLSASDLSVDANTMNFQLLPKLSDHQQGTPQQTLERLHVGHPTLSLQSNDIVCLMAKVDQWDDHAWVLAVDMKNRRLKDVAQFGAERTLGISLVYISSMISEYLRTAPGIKGNLKRQGVVFTVPSHKKQTRMVHLSTPSWKGGDQQNSGTSMDDGEDNMDLDLDMFFG

>OsEDR1-10

MDDLPDDQGQSDAHPSDPNWWSSHAEHKLGSISLTKQERNSGSPGSSHCGEGDHSLWAAQTLWCTGCLSSPIPNGFYSIIPDKKLKERFDTIPSPDDLYSLGIEGFKAEIILVDLEKDKKLSAIKQLCAALVKGLKSNPAAMIKKIAGLVSDFYKRPNPQLSPARTSSEEISHFMENRGVQLLGQIRHGSCRPRAILFKVLADAVGMDCKLLVGIPNEEYHEYDDSSKHMSVVVMLKSVEFLVDLMRFPGQLVPFSSKAIITSHISAAGESDSADYDSCDSPLEPNSPLCSQRQEQDDNNRSFKVPSLRNIMLKSTNSMEGKLRCSSHSEPNVANSFCGRSRRKVVEEHQRTASSSPEHPLSRTRGRSMLGDRQHGDGVAVSRSDGASTSNMRRGRRRSISFTPEIGDDIVSAVRAMSERMRENRLSRGQNDGSPGQLNDSQKNESPHDFNDNELHVRGPDEQEGSRRQVSNQKAVSLPSSPHRLRSDGSGLRGPAEFLTADLMSTWNKVLRSSPFLNKPLLPFEEWHIEFSEITVGTRVGIGFFGEVFRGIWNGTDVAIKLFLEQDLTTENMEDFCNEISILSRLRHPNVILFLGACMKPPHLSLVTEYMEMGSLYYLIHASGQKGKLSWRRRLKMLRDICRGLMCMHRLKIVHRDLKSANCLVNKHWAVKLCDFGLSRVMSNSAMNDNSSAGTPEWMAPELIRNEPFTEKCDIFSLGVIMWELCTLSRPWEGIPSVQVVYNVANEGARLEIPDGPLGSLIADCWAEPDKRPGCQEILTRLLDCEYTLC

>OsEDR1-11

MKHLLRKLHLSGGGGSGGGAAAAGAPSGEHHHRPRQHRRSSAQPPPLPPPPVVAAAAAAEAAPVMAPVAAPVAAAAAEPRGMGADATMTRLEEEYQVRLALAISASDHAGLVDADSVQIRAAERISLGGAAGDRGPMEALSARYWNHCVVNYDERLSDGFYDVCGAPMHPHFQAKFPSLTTLRAVPVGGDAAYVAVLVNRERDPALKRLEGRALAIAAQDRAEHGGVASPELVQKIANLVVDAMGGPVDDADEMNREWGVKSRALCLQRNSIVLPLGLLRIGLSRHRSLLFKVLADRVNLPCKLVKGIYYTGTDEGAINLVKIDFDSVEYIVDLMGAPGTLIPSDISGSQFQDSNNSQLSNDAIEESVAELCIALEQISAGCKNTSDMGGSSSEQKSALALASSQLEDIFHTENPLKQSTISDGGEIPHLMKVNDAPMYLVPTEVDPQFAQNLQDLLLEGTALLPTYEKPEICKHTASEDDKTAGWLVIAKTGQNLPNGHVAEDSPLQHGNTKTLAVVNCFHEDAQHDVGNTEAIGRNLDLHDHTAHAIANEDQRFSEDSLVKMPGSSNGNLDKSSCSSTKTISSVIDDVADYEIPWEDLHIGERIGLGSYGEVYHADWNGTEVAVKKFLDQDLSGVALDQFKCEVGIMSRLRHPNVVLFLGYVTQPPNLSILTEYLPRGSLYRLLHRPNSQIDETRRLKMALDVAKGMNYLHASHPTIVHRDLKSPNLLVDKNWVVKVSDFGMSRLKHHTFLSSKSTAGTVISLMLW

>OsEDR1-12

MSRMKHLLRKLHLSGGGGSGGGAAAAGAPSGEHHHRPRQHRRSSAQPPPLPPPPVVAAAAAAEAAPVMAPVAAPVAAAAAEPRGMGADATMTRLEEEYQVRLALAISASDHAGLVDADSVQIRAAERISLGGAAGDRGPMEALSARYWNHCVVNYDERLSDGFYDVCGAPMHPHFQAKFPSLTTLRAVPVGGDAAYVAVLVNRERDPALKRLEGRALAIAAQDRAEHGGVASPELVQKIANLVVDAMGGPVDDADEMNREWGVKSRALCLQRNSIVLPLGLLRIGLSRHRSLLFKVLADRVNLPCKLVKGIYYTGTDEGAINLVKIDFDSVEYIVDLMGAPGTLIPSDISGSQFQDSNNSQLSNDAIEESVAELCIALEQISAGCKNTSDMGGSSSEQKSALALASSQLEDIFHTENPLKQSTISDGGEIPHLMKVNDAPMYLVPTEVDPQFAQNLQDLLLEGTALLPTYEKPEICKHTASEDDKTAGWLVIAKTGQNLPNGHVAEDSPLQHGNTKTLAVVNCFHEDAQHDVGNTEAIGRNLDLHDHTAHAIANEDQRFSEDSLVKMPGSSNGNLDKSSCSSTKTISSVIDDVADYEIPWEDLHIGERIGLGSYGEVYHADWNGTEVAVKKFLDQDLSGVALDQFKCEVGIMSRLRHPNVVLFLGYVTQPPNLSILTEYLPRGSLYRLLHRPNSQIDETRRLKMALDVAKGMNYLHASHPTIVHRDLKSPNLLVDKNWVVKVSDFGMSRLKHHTFLSSKSTAGTVISLMLCRSGWHQRFYVMSHQMRSVTYTVLE

>OsEDR1-13

MDDTPTSSGKSEVNSCEPSWWPPDFLAKIESVSLSRKQSVFSDKEPRSNLRSSSWKASQLLWSTGTYSGFIPNGFYSIIPDKKLKENFPTIPSLDDLQTLEADGLKADIIIVDVERDKKLFMLKQLSGALVKGLNSSPALVIKKIAGLVFDCFKSLDPDVSPARSPTEDNHFFGNKGSQLLGQIKHGSCRPRAILFKVLADAVGLESKLVVGLPDDGGVGFVDSYKHMSVVVSLNSMELLVDLMRFPGQLIPFSAKAIFISHISAAGESDSAENDSCDSPLEPNSPLYGLSDKVEAEGIEASSNLSGRSLRNVMLRSRTFSEGKLSTSCSEPNIANAFWRRSQRRGVAEEPRGASSSPEHPLMKTRARSILGGEQHSFQEYAESGVTSRSDGLGGASTSKTRRIRGRSISITPEIGDDIVRAVRAMNETLKQNRLQRDHVNEGSPSYVGEDQNNASDCPNNDDTSGGVVATNNGPRNRNGSTQKAMSLPSSPHEYRAQISETINPCDFVSKEKMVLAWNKVLQSSPFLNKPLLPFEEWNIDFSELTIGTRVGIGFFGEVFRGIWNGTDVAIKVFLEQDLTTENMEDFCNEIYILSRLRHPNVILFLGACMVPPHLSMVTEYMEMGSLYYLIHMSGQKKKLSWRRRLKIVRDICRGLMCIHRMKIVHRDLKSANCLVNKHWTVKICDFGLSRVMTDSPMTDNSSAGTPEWMAPELIRNEPFTEKCDIFSLGVIMWELCTLSRPWDGISPVQVVYTVANEGSRLEIPEGPLGKLIADCWAEPQDRPSCQEILTRLLDCEYAVS

>OsEDR1-14

MKADAKWPAMVLGGGGGGGRRASPGSAPPPAAPAAVAYSLLATSPPASIGNGGGSPHCDDGDASRGLGVADWLRLQRHSSGSSAGDDGDGFSSVSTLATADKGGDPADRPAGSSGGGGSKSWAQQAEEAYQLQLALALRLCSEASTAPDPNFLDSAVAAADDHHRDAPSPQSLSHRFWVNGSLSYSDKVLDGFYLIHGMDPFVWTLCNDLRDGARVPSIESLKAMNPTESSVEVVLIDRVVDYDLRQLISTAIDVSRSRADSREITTRLAGIVSSKMGGSVASTEEHELCPRWRDSAGFLKISSGSVVLPIGKLSIGLCRHRSLLFKTLADTISLPCRVVRGCRYCKSAGAASCLVHFGNDREYLIDLIGNPGFLSEPDSLLNGLSSISVSSPLRPPKYNSADIVNNFKSLAKQYFLDCQSLNMMFNDPAAVSGTVVDLDEAMGSNIGPNLSPATNSDFQANFSHRSRGAQSSGQDGNFLIQKSSPEDTQSAQSDPFSDISLDIEDLIIPWSELVLKEKIGAGSFGTVHRADWNGSDVAVKILMEQDFHPERLKEFLREVAIMKSLRHPNIVLFMGAVTQPPKLSIVTEYLSRGSLYRILHKHGARENLDEKRRLSMAFDVAKGMNYLHKRNPPIVHRDLKSPNLLVDKKYTVKVCDFGLSRLKANTFLSSKTAAGTVSSYYRYFYLLIFFQLNRASKCFRCNIL
