# Supplementary material for: Identification and expression analysis of EDR1-like genes in tobacco (Nicotiana tabacum) in response to Golovinomyces orontii
Source: PeerJ. 2018 Jul 10;6:e5244. doi: 10.7717/peerj.5244 (PMC6044316; doi:10.7717/peerj.5244)
Supplement: Supplemental Information 8 [file peerj-06-5244-s008.docx]

| **Supplemental Table 1. The primer sequences used in qRT-PCR for detection the expression patterns of *NtEDR1-like*genes** | | |
| --- | --- | --- |
| Gene | Forward (5' - 3') | Reverse (5' - 3') |
| *NtEDR1-1* | CCAACGACGCTCAATCTA | CCGCCACCAGCACTCACC |
| *NtEDR1-2* | CCATTTATGAGCACCAGCGAAGT | ATCATTACCGCTTGACCTATCT |
| *NtEDR1-3* | GGAAATGAATCTCCCTCTTCTA | TCTTGTCCCTGCTCCTTCGTAA |
| *NtEDR1-4* | TTCATCGGTCCAACAATC | AACCCAGTTCTTATCCACAA |
| *NtEDR1-5* | CTTCCTGCTGGTGGTA | CTCAAACTGCGTGCTT |
| *NtEDR1-6* | TTGTTGCTAATAGAATGGGAGG | AAGACAGCCAAGAGGAAGAATG |
| *NtEDR1-7* | CAGGACAAACTGACCGAAATAA | CTGGAGGGACAGGTAACCGAGA |
| *NtEDR1-8* | GAAAGTGCCCGCATTCCATCTA | ATGATTGCAGACTACCTTAGCG |
| *NtEDR1-9* | CTTGCCAAACAGTATTTCTCAG | TCTCCATATTATTTCGGTCAGT |
| *NtEDR1-10* | CCTGGTTGATTTGGTCGGTAAG | TATCTCGGAAAGCGCAAAGGTG |
| *NtEDR1-11* | ATCGGGTAGTTTATGCTG | GGTCTTTCGTTTGGCTCT |
| *NtEDR1-12* | TGGAGGCAAGAGGTAGAAC | TGAACCAGCACCAACACG |
| *NtEDR1-13* | GGCAGGTGAATGATGAAA | AAGAATCCAATCCCGACA |
| *NtEDR1-14* | GTTACCCTCGTCTCCCAATG | CCCAAAGAATCCAATCCC |
| *NtEDR1-15* | GGTTCGGGAGGGTCGCATAG | ACGAGCCGCAGCCGTCAT |
| *NtEDR1-16* | GTTGGACCAGAGGAGACG | CGACAGACCGAAATCACA |
| *NtEDR1-17* | CCAGATCGGGTAGTTTATGC | TATTCGCAGTCTAGCAGTCG |
| *NtEDR1-18* | AATGGCACGGAACTGAAGTA | TTGAAAGGTTTGGAGGACGA |
| *NtEF1a* | GCATTGCTTGCTTTCACCCTT | AACCTCCTTCACGATTTCATCATACC |
