# Supplementary material for: Identification and expression analysis of EDR1-like genes in tobacco (Nicotiana tabacum) in response to Golovinomyces orontii
Source: PeerJ. 2018 Jul 10;6:e5244. doi: 10.7717/peerj.5244 (PMC6044316; doi:10.7717/peerj.5244)
Supplement: Supplemental Information 9 — The protein sequences of EDR1-like genes in each species is presented in Supplementary Materials (Arabidopsis in File S1; tobacco in File S2; tomato in File S3; and rice in File S4). [file peerj-06-5244-s009.docx]

| **Supplemental Table 2. Summary of the *EDR1-like*genes in different plant species** | | | | | | | |
| --- | --- | --- | --- | --- | --- | --- | --- |
| Species | Gene Name | Locus | Number of  EDR1s | Species | Gene Name | Locus | Number of  EDR1s |
| Arabidopsis | *AtEDR1-1* | At1g08720.1 | 12 | Tobacco | *NtEDR1-1A* | —— | 19 |
|  | *AtEDR1-2* | At5g11850.1 |  |  | *NtEDR1-1B* | —— |  |
|  | *AtEDR1-3* | At1g73660.1 |  |  | *NtEDR1-2* | —— |  |
|  | *AtEDR1-4* | At1g18160.1 |  |  | *NtEDR1-3* | —— |  |
|  | *AtEDR1-5* | At5g03730.1 |  |  | *NtEDR1-4* | —— |  |
|  | *AtEDR1-6* | At5g03730.2 |  |  | *NtEDR1-5* | —— |  |
|  | *AtEDR1-7* | At4g24480.1 |  |  | *NtEDR1-6* | —— |  |
|  | *AtEDR1-8* | At2g31010.2 |  |  | *NtEDR1-7* | —— |  |
|  | *AtEDR1-9* | At2g31010.1 |  |  | *NtEDR1-8* | —— |  |
|  | *AtEDR1-10* | At3g58640.2 |  |  | *NtEDR1-9* | —— |  |
|  | *AtEDR1-11* | At3g58640.1 |  |  | *NtEDR1-10* | —— |  |
|  | *AtEDR1-12* | At2g42640.1 |  |  | *NtEDR1-11* | —— |  |
| Rice | *OsEDR1-1* | Os03g06410.1 | 14 |  | *NtEDR1-12* | —— |  |
|  | *OsEDR1-2* | Os06g12590.1 |  |  | *NtEDR1-13* | —— |  |
|  | *OsEDR1-3* | Os02g50970.1 |  |  | *NtEDR1-14* | —— |  |
|  | *OsEDR1-4* | Os10g29540.1 |  |  | *NtEDR1-15* | —— |  |
|  | *OsEDR1-5* | Os02g12810.1 |  |  | *NtEDR1-16* | —— |  |
|  | *OsEDR1-6* | Os02g32610.3 |  |  | *NtEDR1-17* | —— |  |
|  | *OsEDR1-7* | Os02g32610.2 |  |  | *NtEDR1-18* | —— |  |
|  | *OsEDR1-8* | Os04g52140.1 |  | Tomato | *SlEDR1-1* | Solyc01g097980.3.1 | 8 |
|  | *OsEDR1-9* | Os09g39320.1 |  |  | *SlEDR1-2* | Solyc08g065250.3.1 |  |
|  | *OsEDR1-10* | Os02g14530.1 |  |  | *SlEDR1-3* | Solyc10g083610.2.1 |  |
|  | *OsEDR1-11* | Os02g12810.3 |  |  | *SlEDR1-4* | Solyc10g085570.3.1 |  |
|  | *OsEDR1-12* | Os02g12810.2 |  |  | *SlEDR1-5* | Solyc09g009090.3.1 |  |
|  | *OsEDR1-13* | Os01g48330.1 |  |  | *SlEDR1-6* | Solyc04g076480.3.1 |  |
|  | *OsEDR1-14* | Os02g32610.4 |  |  | *SlEDR1-7* | Solyc07g055130.3.1 |  |
|  |  |  |  |  | *SlEDR1-8* | Solyc02g076780.3.1 |  |

The protein sequences of *EDR1-like*genes in each species is presented in Supplementary Materials (Arabidopsis in Supplementary file 1; tobacco in Supplementary file 2; tomato in Supplementary file 3; and rice in Supplementary file 4).
