# Supplementary material for: Identification and expression analysis of EDR1-like genes in tobacco (Nicotiana tabacum) in response to Golovinomyces orontii
Source: PeerJ. 2018 Jul 10;6:e5244. doi: 10.7717/peerj.5244 (PMC6044316; doi:10.7717/peerj.5244)
Supplement: Supplemental Information 10 — “√” represents protein has transmembrane helices. [file peerj-06-5244-s010.docx]

| **Supplemental Table 3.**  **Prediction of NLS sequences and the existence of transmembrane helices of the tobacco EDR1-like proteins** | | | |
| --- | --- | --- | --- |
| Name | Bipartite NLS | Monopartite NLS | Transmembrane helices |
| NtEDR1-1A | ISKRPSSSNQVDWTSPLAIGTSLYKGGRGP RPPAPMMWKNRYAHNEVPWKNDSDSEALFPKKSCGS | —— | —— |
| NtEDR1-1B | ISKRPSSSNQVDWTSPLAIGTSLYKGGRGP RPPAPMMWKNRYAHNEVPWKNDSDSEALFPKKNCGS | —— | —— |
| NtEDR1-2 | FLKKLHIGSNQSEDSEGSTSSSKSKRLSD | RIMKRLRHPNVVLF | √ |
| NtEDR1-3 | FLKKLHIGSNQSEDSEGSTSSSKSKRLSD | RIMKRLRHPNVVLF | √ |
| NtEDR1-4 | LLKKLHLVPNESVDSEGSTSSAKTKRLSD TAERRSRFRESSTENASPSSSNNSEKQIKAE ERRSRFRESSTENASPSSSNNSEKQIKAEKG | RIMKRLRHPNVVLF | √ |
| NtEDR1-5 | —— | —— | —— |
| NtEDR1-6 | —— | —— | —— |
| NtEDR1-7 | —— | —— | —— |
| NtEDR1-8 | —— | —— | —— |
| NtEDR1-9 | —— | —— | —— |
| NtEDR1-10 | SGRRSSYTLLNQIPDDNFVLPPPPKFSAG FRGKRLDIPSDLNPQVATIIEACWAKCVACT | —— | —— |
| NtEDR1-11 | GQKKRLSWQKRLNMLRDICRGLMCLHRMKI |  | —— |
| NtEDR1-12 | —— | MGAVTKRPHLS | —— |
| NtEDR1-13 | SKFSRIDSGEAPSKRQRV | GEAPSKRQRVALE | —— |
| NtEDR1-14 | —— | —— | —— |
| NtEDR1-15 | SGRRSSYTLLNQIPDDNFVLPPPPKFSAG FRGKRLDIPSDLNPQVATIIEACWAKCVACT | —— | —— |
| NtEDR1-16 | KKLVEQAMLASGEPMLLQECRSTKRLGGS | MGAVTKRPHLS | —— |
| NtEDR1-17 | GQKKRLSWQKRLNMLRDICRGLMCLHRMKI | —— | √ |
| NtEDR1-18 | LLKKLHLMPNESVDSEGSTSSAKTKRLSD TAERRSRFRESSTGNESPSSSNNSEKQIKAE ERRSRFRESSTGNESPSSSNNSEKQIKAEKG | —— | —— |

“√” represents protein has transmembrane helices.
