# Supplementary material for: Identification and expression analysis of EDR1-like genes in tobacco (Nicotiana tabacum) in response to Golovinomyces orontii
Source: PeerJ. 2018 Jul 10;6:e5244. doi: 10.7717/peerj.5244 (PMC6044316; doi:10.7717/peerj.5244)
Supplement: Supplemental Information 11 [file peerj-06-5244-s011.docx]

| **Supplemental Table 4. Putative motifs conserved in the amino acid sequences of the tobacco EDR1-like proteins** | | | |
| --- | --- | --- | --- |
| Motif | Width | Best possible match | Annotated domain |
| 1 | 50 | EFLREVAIMKRLRHPNVVLFMGAVTRPPNLSIVTEYLPRGSLYRLJHRPG | kinase |
| 2 | 50 | IVHRDLKSPNLLVDKNWTVKVCDFGLSRLKHBTFLSSKSTAGTPEWMAPE | kinase |
| 3 | 50 | RNEPSNEKCDVYSFGVILWELATLQQPWSGMNPMQVVGAVGFQGKRLEIP | kinase |
| 4 | 50 | VEECEIPWEDLVLGERIGLGSFGEVYRADWNGTEVAVKKFLEQDFTAESL | kinase |
| 5 | 50 | VLPJGSLSIGLCRHRALLFKVLADIVGLPCRJVKGCKYTGADDAASNLVK | EDR1 |
| 6 | 21 | LDERRRLRMALDVAKGMNYLH |  |
| 7 | 28 | ETVSYRYWVYGCLSYDDKVPDGFYDIYG |  |
| 8 | 29 | LDREYJVDLMGDPGTLIPPDTSINGPSSI |  |
| 9 | 29 | SAEVILVBRRADPKLKELZQRILEJSVGC |  |
| 10 | 41 | DLDPIVADIIEKCWQTDPWLRPSFAZIMAALKPLQKPIVTP | kinase |
